# Recent advances in dermal fibroblast senescence and skin aging: unraveling mechanisms and pioneering therapeutic strategies

**DOI:** 10.3389/fphar.2025.1592596

**Published:** 2025-06-18

**Authors:** Li Nan, Pengchao Guo, Wang Hui, Fang Xia, Chenggang Yi

**Affiliations:** ^1^ Department of Plastic Surgery, The Second Affiliated Hospital of Zhejiang University School of Medicine, Hangzhou, China; ^2^ Emergency Department, The Second Affiliated Hospital of Zhejiang University School of Medicine, Hangzhou, China

**Keywords:** aging, dermal fibroblast, skin, SASP, signaling pathways

## Abstract

Aging is a multifactorial process that affects skin integrity through the progressive decline of dermal fibroblast function. Dermal fibroblasts are key regulators of extracellular matrix (ECM) composition, wound healing, and tissue homeostasis. However, their dysfunction contributes to structural deterioration, chronic inflammation, and impaired regenerative capacity. Cellular senescence, a fundamental characteristic of aging, results in the buildup of senescent fibroblasts that release growth factors, matrix-degrading enzymes, and pro-inflammatory cytokines, known as the senescence-associated secretory phenotype (SASP). This study examines the impact of fibroblast senescence on dermal aging, highlighting mechanisms such as DNA damage, mitochondrial dysfunction, oxidative stress, and telomere attrition. The role of SASP-driven ECM degradation, matrix metalloproteinases (MMPs) activation, and fibroblast-keratinocyte communication breakdown are explored, demonstrating their collective contribution to skin aging. Additionally, key signaling pathways, including p16INK4a/RB, p53, NF-κB, mTOR, and TGF-β, are implicated in fibroblast senescence and chronic inflammation. Recent advancements in therapeutic strategies targeting fibroblast aging, such as senolytics, extracellular vesicle-based interventions, and metabolic reprogramming, offer promising avenues for skin rejuvenation. This review delves into the molecular and cellular dynamics of dermal fibroblast aging, emphasizing their relevance for developing novel anti-aging interventions.

## 1 Introduction

Aging is a multifaceted biological process characterized by the gradual deterioration of cellular and systemic functions, resulting in heightened vulnerability to diseases and an increased risk of mortality (Guo et al., 2022). At the cellular level, one of the hallmarks of aging is cellular senescence, a state of irreversible growth arrest triggered by various stressors, including DNA damage, telomere attrition, oxidative stress, and oncogenic signaling (López-Otín et al., 2023). Senescent cells, while initially beneficial in preventing the propagation of damaged cells, accumulate over time and contribute to chronic inflammation and tissue dysfunction through the release of growth factors, matrix-remodeling enzymes, and pro-inflammatory cytokines, known as the senescence-associated secretory phenotype (SASP) (Wang et al., 2024). This persistent low-grade inflammation, termed inflammaging, exacerbates age-related pathologies such as neurodegeneration, cardiovascular disease, and cancer.

**FIGURE 1:**
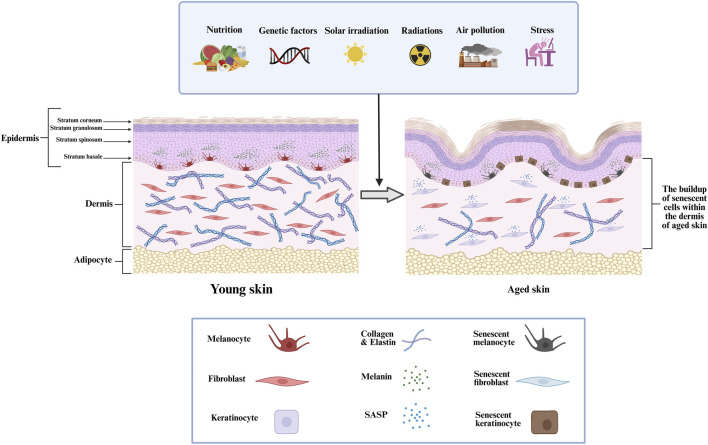
The structural alterations within the dermis that related to skin aging, the progressive increase of senescent cells in aged dermal tissue, and the impact of different intrinsic and extrinsic factors.

Furthermore, mitochondrial dysfunction, epigenetic alterations, and impaired proteostasis also play critical roles in the aging process by disrupting cellular homeostasis (Srivastava, 2017; Wang K. et al., 2022). The skin is the largest organ of the human body, functioning as a highly specialized and dynamic barrier that protects against environmental insults, pathogens, and mechanical trauma while regulating thermoregulation and hydration (Kabashima et al., 2019; Gilaberte et al., 2016). Structurally, it comprises a complex interplay of epithelial, connective, and subcutaneous tissues, each contributing to its protective and homeostatic roles. The outermost layer consists of a continuously regenerating stratified squamous epithelium, primarily composed of keratinocytes, which form a resilient barrier against external stressors. Beneath this, a dense fibroblast-rich connective tissue network provides biomechanical strength and elasticity through extracellular matrix (ECM) components, such as collagen and elastin. The innermost layer is composed of adipose-rich subcutaneous tissue, functioning as an insulator and energy reserve while also aiding in shock absorption and metabolic regulation (Gilaberte et al., 2016; Richardson, 2003; Lee and Kim, 2022). However, intrinsic aging, driven by genetic and cellular factors, along with extrinsic factors such as ultraviolet (UV) radiation, pollution, and oxidative stress, leads to structural and functional deterioration (Farage et al., 2008; Hussein et al., 2025). Aged skin exhibits collagen degradation, reduced fibroblast activity, impaired wound healing, and increased senescence of dermal and epidermal cells, contributing to loss of elasticity, thinning, and the formation of wrinkles (Shin et al., 2019; Chin et al., 2023). The accumulation of senescent cells, particularly in the dermis, exacerbates chronic inflammation through the SASP, further accelerating aging-related skin degeneration (Figure 1) (Konstantinou et al., 2024).

Given that dermal fibroblasts have a pivotal role in managing skin integrity by regulating ECM composition, wound healing, and cellular homeostasis, their dysfunction is a key driver of age-related skin deterioration. In this review, we examine the process of skin aging by focusing on the role of dermal fibroblasts, investigating their functional decline, ECM remodeling, and senescence-associated alterations that contribute to age-related structural and physiological deterioration.

## 2 The characteristics of dermal fibroblasts

Dermal fibroblasts are mesenchymal cells specialized in preserving the skin’s structural framework and functional homeostasis. As the predominant cell type in the dermis, fibroblasts are responsible for the remodeling, synthesis, and degradation of the ECM, which provides the skin with mechanical strength, elasticity, and hydration (Plikus et al., 2021; Thulabandu et al., 2018). These cells primarily produce collagen (types I and III), glycosaminoglycans (GAGs), fibronectin, and elastin, ensuring the stability of the dermal-epidermal junction and facilitating skin repair and regeneration (Boraldi et al., 2024). In addition to their structural role, fibroblasts act as key regulators of skin homeostasis by modulating various cellular and biochemical processes. They secrete growth factors and cytokines such as platelet-derived growth factor (PDGF), fibroblast growth factors (FGFs), insulin-like growth factor-1 (IGF-1), and transforming growth factor-beta (TGF-β), which influence keratinocyte proliferation, immune responses, and angiogenesis (Faria and Andrade, 2024; Zhao et al., 2019). Fibroblasts also communicate with immune cells, endothelial cells, and epidermal keratinocytes through paracrine signaling, contributing to the regulation of skin inflammation and tissue repair (Plikus et al., 2021; Dong et al., 2024). Mechanotransduction is another crucial function of fibroblasts, as they respond to mechanical stimuli from their microenvironment and modulate ECM production accordingly. Fibroblasts sense and adapt to mechanical forces via integrins and focal adhesion complexes, which regulate their morphology and behavior (Junker, 2024; Di et al., 2023). This dynamic ability to respond to environmental cues ensures the maintenance of wound healing processes and tissue integrity. In healthy skin, dermal fibroblasts typically remain inactive; however, following injury, they undergo activation and differentiate into myofibroblasts. These myofibroblasts gain increased contractility and contribute to wound closure by synthesizing extracellular matrix proteins (Sierra-Sánchez et al., 2021). However, excessive fibroblast activation can lead to fibrosis, characterized by excessive collagen deposition and ECM stiffening, contributing to pathological conditions such as scleroderma and hypertrophic scarring (Wang S. et al., 2022).

## 3 The role of senescent dermal fibroblasts in skin aging

Aging is associated with a progressive decline in dermal fibroblasts, leading to impaired skin structure, decreased regenerative capacity, and ECM degradation. Studies have shown that the total fibroblast population in human skin declines significantly with age, contributing to the thinning of the dermis, reduced collagen production, and compromised wound healing (Kühnel et al., 2025; Varani et al., 2006). This reduction is primarily driven by cellular senescence, apoptosis, and decreased proliferative potential, which collectively weaken the functional and structural integrity of the skin. An investigation was conducted to examine the age-related decline in dermal fibroblast number using single-cell RNA sequencing (scRNA-seq) and computational transcriptomic analysis on over 5,000 dermal fibroblasts isolated from sun-protected human skin (Solé-Boldo et al., 2020). The study employed cell clustering techniques and differential gene expression analysis to identify distinct fibroblast subpopulations and assess changes in their abundance with aging. The findings showed a significant reduction in fibroblast density, which disrupts ECM homeostasis and compromises dermal integrity. This decline is attributed to diminished proliferative capacity, increased cellular senescence, and impaired self-renewal mechanisms. Additionally, cell-cell interaction mapping revealed weakened fibroblast-keratinocyte communication in aged skin, further exacerbating fibroblast depletion and contributing to structural deterioration (Solé-Boldo et al., 2020). Another study showed that a histological analysis of sun-protected skin from young (18–29 years) and old (80+ years) individuals demonstrated a 35% reduction in fibroblast density in aged skin. This decline was accompanied by a 68% reduction in type I procollagen content and a 30% decrease in fibroblast collagen-synthetic capacity, indicating that both fibroblast loss and impaired fibroblast function contribute to the decline in ECM homeostasis (Fligiel et al., 2003). Additionally, fibroblasts in aged skin exhibited reduced mechanical interactions with collagen fibers, leading to impaired mechanotransduction and further compromising their ability to maintain dermal structure (Fligiel et al., 2003; Fisher et al., 2016).

**FIGURE 2:**
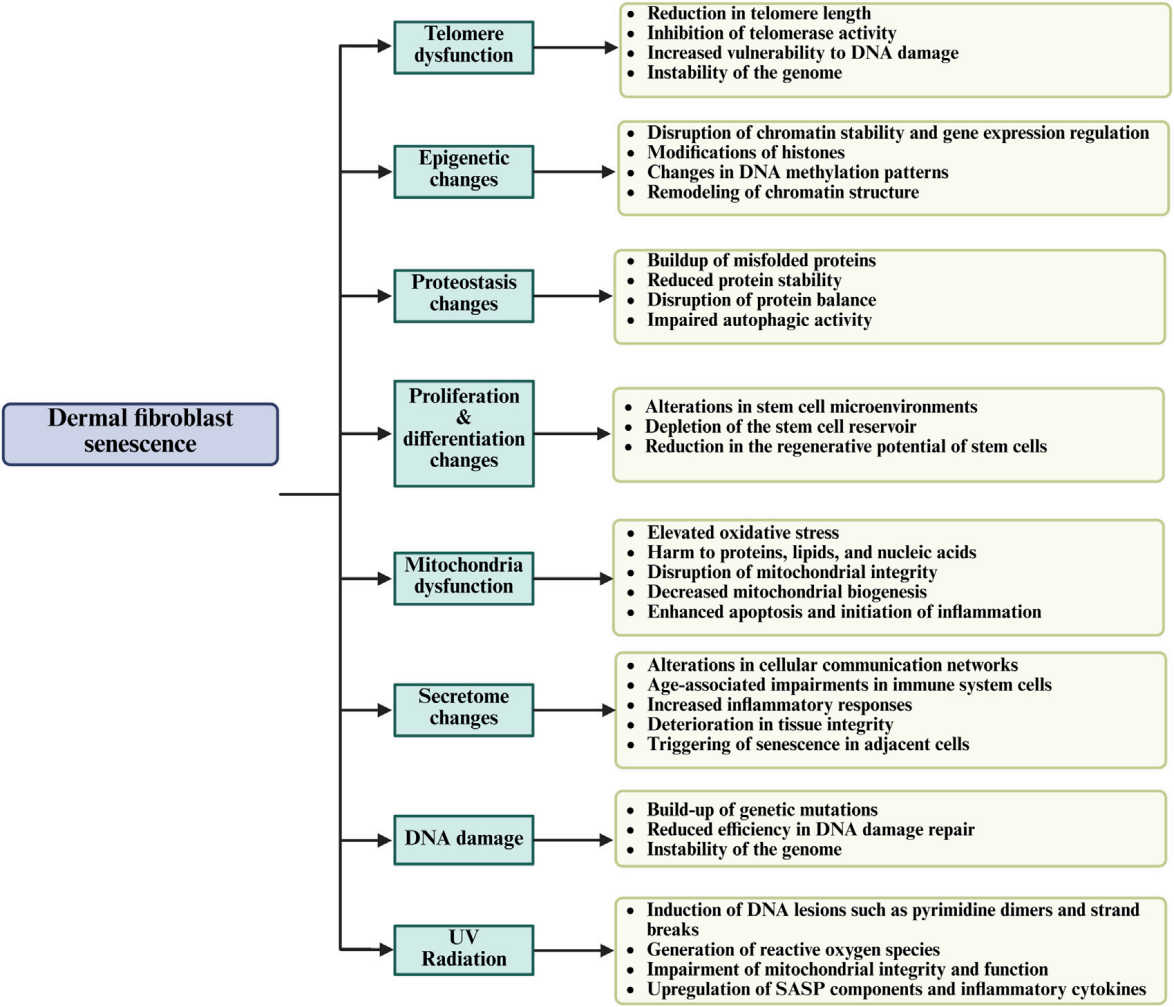
The general mechanisms of dermal fibroblast senescence and their effect on skin aging through the regenerative capacity, impairment of cellular function, and tissue integrity.

Cellular senescence is initiated by multiple stimuli, including oxidative stress (reactive oxygen species (ROS)), DNA damage, ionizing radiation, telomere attrition, and mitochondrial dysfunction (Figure 2) (Torres et al., 2024). These stressors activate signaling pathways that upregulate cyclin-dependent kinase (CDK) inhibitor proteins, including p16INK4a and p21CIP1/WAF1. The increased expression of these inhibitors suppresses CDK activity, leading to hypophosphorylation of the retinoblastoma (RB) protein and ultimately triggering a sustained G1-phase cell cycle arrest (Kudlova et al., 2022; Jin et al., 2024). Additionally, activation of p53 (TP53) in response to DNA damage contributes to the upregulation of p21, reinforcing senescence onset (Jin et al., 2024; Mijit et al., 2020). Furthermore, senescence-associated β-galactosidase (SA-β-Gal) is among the most commonly utilized markers for identifying senescent cells. It is detected histochemically at pH 6.0 due to increased lysosomal activity. Recent findings suggest that SA-β-Gal activity correlates with enhanced lysosomal biogenesis and metabolic dysfunction in senescent fibroblasts, reinforcing its role as a key senescence biomarker (Franco et al., 2025). Unlike apoptotic cells, senescent fibroblasts evade immune clearance and persist within the dermal microenvironment, where they actively secrete a pro-inflammatory and tissue-degrading SASP. The aberrant accumulation of senescent fibroblasts leads to progressive loss of cellular identity, which causes altered gene expression profiles, impaired ECM remodeling, and dysregulated signaling pathways essential for maintaining dermal homeostasis (Pereira et al., 2019; Song et al., 2020).

Recent research has introduced nuanced mechanisms that extend beyond classical senescence, enriching our understanding of how fibroblast aging contributes to skin degeneration. Among these, inflammaging and senescent drift have garnered attention for their systemic and temporal implications. Rather than acute, pathogen-driven inflammation, the skin of elderly individuals experiences a chronic inflammatory bias, shaped by long-term accumulation of senescent fibroblasts and their persistent secretion of SASP factors (Pilkington et al., 2021). Unlike acute inflammation, which is self-resolving and beneficial, inflammaging perpetuates tissue damage, promotes ECM degradation, and impairs cutaneous immune responses (Pilkington et al., 2021). Recent studies indicate that fibroblast-derived extracellular vesicles may serve as amplifiers of this chronic inflammation by disseminating pro-inflammatory signals throughout the dermal microenvironment, establishing a feed-forward loop that exacerbates aging phenotypes and delays wound repair Recent studies indicate that fibroblast-derived extracellular vesicles may serve as amplifiers of this chronic inflammation by disseminating pro-inflammatory signals throughout the dermal microenvironment, establishing a feed-forward loop that exacerbates aging phenotypes and delays wound repair (Xie et al., 2025; Bian et al., 2022; Lyamina et al., 2023). Moreover, inflammaging alters the immunological landscape of the skin, increasing the infiltration of dysfunctional macrophages and CCR2+ monocytes, which further suppress immune surveillance and potentiate age-associated dermopathologies (Fulop et al., 2023). Another evolving concept is senescent drift, referring to the phenotypic and functional heterogeneity that emerges among senescent cells over time. Unlike the uniform senescence response seen in acute stress models, aged dermal fibroblasts exhibit variable transcriptional and secretory profiles influenced by cumulative environmental exposure, epigenetic modifications, and stochastic factors (Sun, 2023). A key mechanistic insight into this heterogeneity was demonstrated by Song et al. who identified carnitine acetyltransferase (CRAT) as a central metabolic regulator whose deficiency in dermal fibroblasts induced mitochondrial dysfunction, oxidative stress, and a switch from oxidative phosphorylation to glycolysis (Song et al., 2023). This metabolic imbalance triggered persistent SASP expression through activation of the cGAS-STING-NF-κB axis, mimicking senescence phenotypes and promoting chronic inflammation and ECM degradation. Their *in vivo* model using fibroblast-specific CRAT-knockout mice confirmed the presence of pronounced aging features, increased SASP factors, and decreased dermal collagen density, all hallmarks of senescent drift (Song et al., 2023). Additionally, Smirnova et al. emphasized that extracellular vesicles derived from mesenchymal stem cells (MSCs), often explored as regenerative agents, can paradoxically contribute to senescent drift if derived from aged or stressed donors. These EVs carry a complex cargo of cytokines, lipids, and nucleic acids capable of propagating senescence to surrounding cells, exacerbating inflammaging and altering the local immune milieu. The authors stress that such secretomes may lack consistency, and without rigorous screening, pose a risk of unintentionally amplifying senescence-related dysfunction (Smirnova et al., 2023). Single-cell RNA sequencing and trajectory analysis have further revealed that fibroblast subtypes transition through intermediate states during aging, indicating that senescence is not a binary switch but a spectrum of progressive cellular states (Tao et al., 2024; Uyar et al., 2020). This insight challenges current therapeutic strategies that broadly target senescence and underscore the need for precision interventions tailored to fibroblast subpopulations.

### 3.1 The impact of the senescence-associated secretory phenotype on skin aging

The SASP encompasses a diverse array of bioactive molecules, including growth factors, microRNAs (miRNAs), cytokines, chemokines, matrix metalloproteinases (MMPs), and small-molecule metabolites, secreted by senescent cells (Wang et al., 2024; Coppé et al., 2010). These factors alter the proliferation and migration of nearby non-senescent cells while also exhibiting immunomodulatory functions (Coppé et al., 2010). Among the key SASP components, pro-inflammatory proteins including interleukin-8 (IL-8), IL-15, IL-1β, and interferon-gamma (IFNγ), along with MMPs involved in ECM degradation (MMP1, MMP3, MMP10, MMP14, etc.), have been identified in both skin aging-associated secretory phenotype (SAASP) and canonical SASP, indicating common senescent signatures across different tissues (Khavinson et al., 2022; Malaquin et al., 2016). Additionally, SAASP exhibits distinct protein expression patterns related to metabolic regulation and adherens junction interactions, highlighting unique molecular adaptations specific to senescent skin fibroblasts (Waldera Lupa et al., 2015; Lopes-Paciencia et al., 2019). A study by Narzt et al. suggests that lipid-derived SASP components contribute to senescence persistence and skin aging by identifying lysophosphatidylcholines (LysoPCs) as key SASP factors in senescent dermal fibroblasts, which promote immune evasion and chronic inflammation through altered chemokine secretion and macrophage signaling (Narzt et al., 2021). In addition, Waldera Lupa et al. performed a systematic molecular investigation of normal human dermal fibroblasts obtained from intrinsically aged skin. The findings reveal that fibroblasts from aged donors exhibit increased nuclear foci positive for promyelocytic leukemia protein and p53 binding protein 1, resembling DNA segments with chromatin alterations reinforcing senescence. Despite the absence of increased DNA damage or telomere shortening, these aged fibroblasts develop a SAASP, distinct from the classical SASP. A total of 998 secreted proteins were identified, with 70 proteins showing age-dependent secretion patterns; suggesting that SAASP is essential in intrinsic skin aging by altering the extracellular environment and fibroblast function (Waldera Lupa et al., 2015).

### 3.2 SASP-driven ECM degradation and MMP activation

The degradation of the ECM in aged fibroblasts results in diminished dermal resilience and reduced thickness, leading to skin laxity and wrinkle formation. The MMPs within the SASP play a crucial role in this process by directly cleaving collagen fibrils, and accelerating ECM breakdown during aging (Feng et al., 2024; Freitas-Rodríguez et al., 2017). The study by Quan et al. investigates the impact of collagen fragmentation and MMPs on dermal fibroblast function in photodamaged human skin. Their findings reveal that chronic UV exposure elevates multiple MMPs, leading to progressive degradation of the ECM, impaired collagen homeostasis, and reduced type I collagen production, which collectively contribute to skin aging. Additionally, fragmented collagen microenvironments were found to disrupt fibroblast function, promoting increased MMP expression and decreased collagen synthesis, highlighting a key mechanism in photoaging and dermal degeneration (Quan et al., 2013). Qin et al. further revealed that the age-associated decrease in dermal fibroblast size independently elevates MMP expression by activating the AP-1 transcription factor complex (c-Fos/c-Jun), thereby facilitating collagen breakdown (Qin et al., 2017). Liu et al. examine the effects of human umbilical cord mesenchymal stromal cell-derived extracellular vesicles (hucMSC-EVs) on dermal fibroblasts during wound healing, identifying a distinct MMP13+ fibroblast subtype that exhibits fetal-like characteristics and has a crucial role in ECM reorganization by expressing MMP13, MMP9, and HAS1, facilitating fibroblast migration and keratinocyte interactions through the PIEZO1-calcium-HIF1α-VEGF-MMP13 signaling pathway, emphasizing the essential role of MMPs in fibroblast-driven ECM remodeling and skin regeneration (Liu Y. et al., 2024). Additionally, Song et al. investigate how UV irradiation induces MMP-1 expression in human dermal fibroblasts through epigenetic regulation. Their findings reveal that UV exposure downregulates carnitine acetyltransferase (CRAT) via promoter hypermethylation, leading to the activation of ERK, JNK, and p38 MAPK signaling pathways, which in turn upregulate MMP-1 expression and accelerate ECM degradation. Overexpression of CRAT mitigated this effect, suggesting that epigenetic modulation of CRAT could serve as a potential therapeutic target for preventing UV-induced skin aging (Song et al., 2024). Furthermore, Asharaf et al. investigate the photoprotective effects of sulfated mannogalactan (BVP-2) from *Bacillus velezensis* in preventing UV-A-induced MMP upregulation and ECM degradation in human dermal fibroblasts. Results indicate that UV-A exposure significantly increases MMP-2, MMP-9, and MMP-1 expression, leading to collagen degradation and photoaging, whereas treatment with BVP-2 downregulates MMP expression by 30%–50%, reducing oxidative stress and ECM breakdown (Asharaf et al., 2024). Molecular docking studies further support that BVP-2 directly interacts with MMPs, inhibiting their enzymatic activity, and highlighting its potential as a natural anti-photoaging agent (Asharaf et al., 2024). Furthermore, Novotná et al. explored the potential of natural bioactive compounds in inhibiting MMP activity and preventing ECM degradation in skin aging, examining the effects of rhamnose, rutinose, hesperidin, and hesperetin on normal human dermal fibroblasts and demonstrating that MMP-2 and MMP-1 levels were significantly decreased, particularly with rutinose and rhamnose, which also enhanced collagen I production, suggesting that these flavonoids and carbohydrates play a protective role in fibroblast-mediated ECM homeostasis, making them promising candidates for cosmetic and dermatological applications to counteract fibroblast dysfunction and collagen breakdown (Novotná et al., 2023). Nevertheless, Yokose et al. investigate MMPs in UVB-induced skin aging, highlighting tissue inhibitor of metalloproteinases-1 (TIMP-1) as a key regulator that suppresses MMP-12, MMP-9, MMP-1, and MMP-3 to prevent collagen and elastic fiber degradation. TIMP-1 overexpression preserves ECM integrity, while its inhibition exacerbates MMP-driven collagen breakdown and inflammation, accelerating skin aging (Yokose et al., 2012).

### 3.3 Altered ECM in skin cancer progression

ECM disruption caused by senescent dermal fibroblasts has been increasingly recognized as a contributing factor to the initiation and progression of skin cancers. These fibroblasts exhibit altered expression of structural proteins and matrix-remodeling enzymes, leading to a microenvironment that supports carcinogenesis. Notably, the excessive activity of MMPs, particularly MMP-1 and MMP-3, degrades type I and III collagen, reducing dermal integrity and facilitating the migration of pre-malignant keratinocytes (Cole et al., 2018). Several studies have shown that senescent fibroblasts influence neighboring epithelial cells not only via soluble SASP factors but also by altering ECM mechanics and biochemistry. Fisher et al. reported that the senescence-driven ECM microenvironment in aged skin impairs keratinocyte-fibroblast interactions, enhancing susceptibility to basal cell and squamous cell carcinoma (Fisher et al., 2023). Additionally, Mavrogonatou et al. demonstrated that extracellular vesicles released by senescent fibroblasts carry ECM fragments and bioactive molecules that modulate keratinocyte behavior and gene expression, favoring transformation (Mavrogonatou et al., 2023). Quan et al. investigate the role of MMP1 in dermal aging and skin tumor susceptibility using a humanized transgenic mouse model that selectively expresses human MMP1 in fibroblasts, revealing that elevated MMP1 activity in fibroblasts leads to collagen fibril fragmentation, disrupted fibroblast-ECM interactions, and reduced fibroblast spreading, which impairs collagen homeostasis and accelerates dermal aging, demonstrating that MMP1-induced ECM degradation creates a pro-inflammatory and tumorigenic microenvironment, significantly increasing susceptibility to skin papilloma formation, highlighting MMP1 as a pivotal factor in age-related dermal degeneration and tumorigenesis (Quan et al., 2023).

UV radiation further amplifies ECM alterations. Kang et al. showed that UVB-irradiated senescent fibroblasts increase fibronectin deposition and activate PI3K/AKT and ERK signaling in HaCaT cells, resulting in enhanced proliferation, a hallmark of early neoplastic transformation (Kang et al., 2008). Likewise, Ezure et al. identified that senescent fibroblast-derived complement factor D negatively regulates COL1A1 expression in nearby fibroblasts, contributing to a collagen-deficient dermal matrix permissive to malignant progression (Ezure et al., 2019). In the context of melanoma, the stromal contribution to tumor development has also been observed. Sadangi et al. reported that senescent fibroblasts and altered ECM components help transformed melanocytes bypass oncogene-induced senescence, a key step in early melanoma formation (Sadangi et al., 2022). Toutfaire et al. showed that senescent dermal fibroblasts, particularly those induced by UVB or replicative aging, exhibit a pro-tumorigenic secretory profile, including elevated MMPs and cytokines that remodel the ECM. While their direct impact on cutaneous squamous cell carcinoma (cSCC) cell behavior was limited, cSCC cells reinforced fibroblast senescence and SASP expression via NF-κB activation. This bidirectional crosstalk promotes ECM degradation and inflammation, creating a microenvironment conducive to tumor progression (Toutfaire et al., 2018). Furthermore, age-related ECM changes not only affect cancer initiation but also shape tumor progression and therapeutic response. The degradation of basement membrane components and disorganization of collagen fibers reduce physical barriers to invasion and support epithelial-mesenchymal transition (EMT), a process critical for cancer cell dissemination (Pretzsch et al., 2022). Altered ECM stiffness and composition, including the accumulation of non-fibrillar collagens and proteoglycans, have been shown to modulate integrin signaling, enhance mechanotransduction, and promote invasive phenotypes in skin cancer cells (Blokland et al., 2020; Zhang J. et al., 2024).

### 3.4 SASP cytokines and chemokines in dermal fibroblasts

In dermal fibroblasts, SASP components, particularly cytokines and chemokines, contribute to chronic inflammation, ECM degradation, and impaired tissue regeneration. These secreted factors sustain autocrine senescence within fibroblasts and act in a paracrine manner, propagating senescence in neighboring cells and exacerbating tissue dysfunction (Table 1) (Acosta et al., 2008; Wlaschek et al., 2021; Li X. et al., 2023). IL-1β, tumor necrosis factor-alpha (TNF-α), and IL-6 are the primary cytokines secreted by senescent fibroblasts, which serve as central mediators of chronic low-grade inflammation in aging skin (Li X. et al., 2023). IL-6 is highly upregulated in senescent fibroblasts and functions through the JAK/STAT3 signaling pathway, reinforcing fibroblast senescence and inducing paracrine senescence in surrounding cells. IL-1β, a potent activator of MMPs, promotes ECM remodeling by upregulating MMP-1, MMP-3, and MMP-9, leading to collagen degradation and dermal thinning. Similarly, TNF-α, a key inflammatory regulator, activates NF-κB and AP-1 signaling, driving fibroblast dysfunction and accelerating ECM breakdown. A study explores the role of EVs secreted by senescent dermal fibroblasts in regulating epidermal homeostasis and inflammation (Choi et al., 2020). Senescent fibroblasts exhibit increased EV production due to elevated dysfunctional lysosomal activity, oxidative stress, and neutral sphingomyelinase (nSMase) activity. Compared to EVs from young fibroblasts, senescent fibroblast-derived EVs impair keratinocyte differentiation and barrier function, while also increasing pro-inflammatory cytokine IL-6 levels, contributing to chronic inflammation in aging skin (Choi et al., 2020). Another study investigates the impact of SASP cytokines and EVs in senescent dermal fibroblasts, highlighting the senomorphic effects of Haritaki fruit extract (Bogdanowicz et al., 2023). Senescent fibroblasts triggered by ionizing radiation exhibited elevated IL-6, IL-1β, and IL-8, contributing to chronic inflammation and ECM degradation (Bogdanowicz et al., 2023). Haritaki extract effectively suppressed SASP cytokine secretion and EV-mediated inflammatory signaling, demonstrating its potential to attenuate fibroblast senescence and delay skin aging (Bogdanowicz et al., 2023). In addition, a study examined the effects of medicinal plant extracts on etoposide-induced senescent dermal fibroblasts, revealing that senescent cells displayed a distinct SASP profile with increased IL-6 secretion, further driving inflammation and tissue degradation. While quercetin and goldenrod extracts demonstrated senolytic properties, selectively reducing the senescent fibroblast burden, chamomile extract unexpectedly amplified IL-6 secretion, intensifying inflammatory responses (Imb et al., 2024). Charoensin et al. investigated the protective effects of nuciferine, an alkaloid from *Nelumbo nucifera*, against H_2_O_2_-induced senescence in human dermal fibroblasts, with particular emphasis on the modulation of SASP cytokines. Oxidative stress from H_2_O_2_ exposure led to increased cytokines secretion, contributing to ECM degradation and chronic inflammation, whereas nuciferine treatment significantly reduced SASP cytokine expression and senescence-associated β-galactosidase activity, suggesting its potential to mitigate fibroblast senescence and inflammation-driven skin aging (Charoensin and Weera, 2022). Ogata et al. explored the role of SASP factors in macrophage dysfunction and its contribution to senescent fibroblasts accumulation in the dermis. SASP factors, particularly IL-1α and GM-CSF, impair both apoptosis induction and phagocytosis by downregulating TNF-α expression and reducing the engulfment capacity of macrophages, leading to an increased burden of senescent fibroblasts in aged and UV-exposed skin (Ogata et al., 2021).

**TABLE 1:** Cytokines and chemokines in SASP of senescent dermal fibroblasts.

Cytokines/Chemokines	Role in SASP	Signaling pathways involved	Biological activity and delivery modality	References
IL-6	Promotes chronic inflammation, tissue remodeling, and immune modulation	JAK-STAT, NF-κB, MAPK.	Soluble and EV-associated; co-acts with IL-8, enhances paracrine senescence	Tanaka et al. (2014), Swaroop et al. (2024), Kolář et al. (2012), Johnson et al. (2020)
TNF-α	Drives inflammation and cell stress responses	TNF-R, NF-κB, MAPK.	Soluble; synergizes with IL-1β and IL-6 in promoting SASP.	Mavrogonatou et al. (2018), Bashir et al. (2009)
TGF-β	Regulates fibrosis and wound healing	TGF-β/SMAD, MAPK.	Soluble and EV-encapsulated; delivered with ECM-related proteins	Liu et al. (2016), Urban et al. (2023)
IL-1α	Initiates inflammation and SASP signaling	NF-κB, inflammasome	Membrane-bound and soluble; primes SASP release	Boxman et al. (1996), Koskela von Sydow et al. (2016), Maret et al. (2004)
IL-1β	Induces inflammation and MMP activation	IL-1R, NF-κB, inflammasome	Soluble; often released via EVs and inflammasome activity	Wang et al. (2024), Wlaschek et al. (2021)
IL-8	Promotes neutrophil chemotaxis and MMP induction	NF-κB, PI3K-Akt, MAPK.	Soluble and EV-encapsulated; co-packaged with CCL2 and IL-6 in EVs	Kolář et al. (2012), Zhang M. et al. (2024)
CCL2	Attracts macrophages and enhances immune infiltration	CCR2, NF-κB, PI3K-Akt	Soluble and EV-associated; facilitates monocyte recruitment and immunosuppression	Chambers et al. (2021), Ohgo et al. (2015)
CCL3	Stimulates T-cell and monocyte chemotaxis	CCR1, NF-κB, MAPK.	Soluble; acts in combination with CCL5 in immune cell recruitment	Wang et al. (2024), Lei et al. (2023)
CCL5	Sustains immune cell infiltration and inflammation	CCR5, MAPK, NF-κB	Soluble and EV-associated; interacts with IL-8 in sustained inflammation	Wang et al. (2024), Lei et al. (2023)
CXCL1	Promotes neutrophil infiltration and inflammatory amplification	CXCR1/2, MAPK, PI3K-Akt	Soluble; part of EV cargo in senescent fibroblasts and UV-induced skin	Kolář et al. (2012), Koczkowska et al. (2025)
CXCL2	Enhances neutrophil recruitment, chronic inflammation	CXCR2, NF-κB, PI3K-Akt	Soluble and EV-bound; upregulated by oxidative stress and photoaging triggers	Bogdanowicz et al. (2023), Kita et al. (2024)

In addition to cytokines, senescent fibroblasts secrete chemokines such as CXCL8, CCL2, and CXCL1, which modulate immune cell infiltration and inflammatory responses in aging skin (Chen et al., 2024). CXCL8 enhances neutrophil recruitment and MMP activity, contributing to collagen degradation and loss of skin elasticity. CCL2, CCL2, or monocyte chemoattractant protein-1 (MCP-1), attracts macrophages to senescent fibroblast-rich areas, increasing chronic immune activation and fibrosis. In addition, CXCL1, a keratinocyte-derived chemokine, has been implicated in fibroblast-keratinocyte signaling dysregulation, leading to delayed wound healing and barrier dysfunction in aged skin (Chen et al., 2024; Esterly and Zapata, 2024). Li et al. studied the protective effects of a fermented rice product called maifuyin, and its bioactive components, choline and succinic acid (SA), against UVA-induced senescence in human dermal fibroblasts. UVA exposure elevated CXCL2 expression, promoting inflammation and ECM degradation, while Maifuyin and SA treatment suppressed CXCL2 secretion, β-galactosidase activity, and MMP-1 expression, highlighting their potential as anti-photoaging agents targeting oxidative stress and chemokine signaling (Li et al., 2025). Fang et al. systematically compared the molecular characteristics of senescent human dermal fibroblast models, highlighting the elevated expression of chemokines CXCL1, CXCL8, and CCL2 (Fang et al. 2024). UVB-induced and atazanavir-treated fibroblasts exhibited the highest SASP-related chemokine expression, creating a pro-inflammatory microenvironment that activates T cells, macrophages, and NK cells. Single-cell RNA sequencing revealed similarities between senescent fibroblasts and aged skin conditions, suggesting that targeting fibroblast-derived chemokines could provide therapeutic strategies for age-related and inflammatory skin diseases (Fang et al. 2024). Smith & Carroll further investigated the role of mTORC1 activation and lysosomal dysfunction in senescent dermal fibroblasts, and they found that elevated IL-6, IL-8, CXCL1, and MMP expression in senescent fibroblasts exacerbates immune dysregulation and tissue remodeling, accelerating skin aging (Smith and Carroll, 2024). Chambers et al. demonstrate that senescent dermal fibroblasts contribute to age-related immune suppression by upregulating CCL2, driving CCR2+CD14^+^ monocyte infiltration and PGE2-mediated T-cell inhibition. p38 MAPK inhibition suppressed CCL2 expression, reduced monocyte recruitment, and restored antigen-specific immunity, highlighting CCL2 as a potential target for improving skin immune function in aging (Chambers et al., 2021). Horn et al. investigated the role of CCL2, CXCL1, and IL-8 in sulfur mustard (SM)-induced senescence in human dermal fibroblasts, revealing their contribution to chronic inflammation and impaired wound healing (Horn et al., 2022). Gene expression analysis and cytokine profiling showed that senescent fibroblasts upregulate CCL2, promoting monocyte recruitment, while CXCL1 enhances neutrophil infiltration, and IL-8 sustains the inflammatory microenvironment, exacerbating tissue damage and delayed regeneration (Horn et al., 2022).

## 4 Signaling pathways involved in dermal fibroblast senescence

Fibroblast senescence in the dermis is a complex and multifaceted process governed by various signaling pathways that contribute to skin aging and declining skin function. The DNA damage response (DDR) pathway serves as an initial defense mechanism against cellular damage induced by oxidative stress, UV radiation, and different genotoxic factors (Huang and Zhou, 2020). Upon sensing DNA damage, DDR proteins including ATM (ataxia-telangiectasia mutated), p53, and p21 are activated to induce cell cycle arrest, therefore preventing the propagation of damaged DNA and promoting repair mechanisms to maintain genomic integrity (Gruber et al., 2013). However, persistent activation of the DDR pathway, due to continuous stress, leads to a state of irreversible cell cycle arrest, a hallmark of senescence (Ma and Zhou, 2025). In dermal fibroblasts, this persistent DDR activation contributes significantly to the aging process by impairing the proliferation of fibroblasts and disrupting the maintenance of the ECM, ultimately accelerating the aging of the skin (Wlaschek et al., 2021; Gruber et al., 2013; Shackelford et al., 2001; Ohtsuka et al., 2004). Zhang et al. presented that MSC-EVs protect fibroblasts from UVB-induced photoaging by modulating the TIMP1/Notch1 pathway, reducing ROS accumulation, DNA damage, and SASP-mediated inflammation, thereby preserving fibroblast viability and ECM integrity (Zhang H. et al., 2024). In contrast, a study identified ATR kinase activation as a key mediator of psoralen-induced fibroblast senescence, where telomeric DNA damage and persistent γ-H2AX foci drive cell-cycle arrest, reinforcing the role of ATR in telomere-dependent senescence (Hovest et al., 2006). Furthermore, MAPK pathways, particularly p38 and JNK, further amplify senescence by promoting SASP-mediated inflammation and ECM degradation, while Notch1 and EGFR/Akt signaling serve as protective regulators, mitigating oxidative stress and sustaining fibroblast survival (Tivey et al., 2013). Mavrogonatou et al. found that UVB-induced fibroblast senescence is regulated through the JNK/ATM-p53 signaling axis, with additional cytoprotective roles of the EGFR/Akt and Nrf2 pathways in stress adaptation and SASP modulation (Mavrogonatou et al., 2022). Tiemann et al. linked ABCC6 deficiency in PXE fibroblasts to premature senescence via a p21-mediated mechanism, independent of p53 activation, leading to increased IL-6 and MCP-1 secretion, suggesting a pro-inflammatory SASP (Tiemann et al., 2020). Furthermore, Frediani et al. identified long non-coding RNAs (lncRNAs) H19 and PURPL as key regulators of fibroblast senescence, with H19 promoting autophagy and senescence through PI3K/AKT/mTOR activation, whereas PURPL inhibition reversed senescence by downregulating p53 (Frediani et al., 2024). Promjantuek et al. explored the involvement of SIRT1 in dermal fibroblast immortalization, showing that SIRT1 activation extends fibroblast lifespan by modulating telomerase activity and repressing p53 signaling (Promjantuek et al., 2022). Additionally, galangin-induced activation of SIRT1 attenuates UVB-induced fibroblast senescence through the deacetylation of p53, decreased expression of SASP markers, and restoration of dermal homeostasis (Wen et al., 2024). Adding more, Haj et al. identified cGAS-STING activation as a key driver of premature senescence in ataxia-telangiectasia (A-T) dermal fibroblasts, marked by an interferon-stimulated gene (ISG) signature independent of interferon expression. Transcriptomic analysis revealed dysregulated ECM remodeling and SASP-associated gene expression, contributing to fibrotic remodeling and cellular dysfunction (Haj et al., 2023).

Another critical regulator of fibroblast senescence is the p16INK4a/pRB pathway, which is essential for maintaining cell cycle control. P16INK4a acts as a potent inhibitor of cyclin-dependent kinases (CDKs), which are essential for phosphorylating the retinoblastoma protein (pRB) (Serizawa, 1998; Ohtani et al., 2004). This phosphorylation event is necessary for progression through the G1 phase of the cell cycle. In senescent fibroblasts, elevated levels of p16INK4a block the phosphorylation of pRB, thus maintaining pRB in its active form and inducing G1 arrest. This arrest prevents fibroblasts from re-entering the cell cycle, contributing to the loss of proliferative capacity that characterizes aging fibroblasts (Vandenberk et al., 2011; Weebadda et al., 2005). The accumulation of p16INK4a in aging skin is associated with reduced fibroblast function and impaired wound healing, which further exacerbates the aging phenotype (Safwan-Zaiter et al., 2022; Adamus et al., 2014). In an effort to identify potential therapeutic targets, Takaya et al. examined the role of Secreted Frizzled-Related Protein 4 (SFRP4) in fibroblast senescence and SASP regulation. Their findings indicate that SFRP4 expression is significantly upregulated in p16INK4a-positive fibroblasts and promotes senescence by enhancing IL-6, IL-8, MMP3, and TNF-α expression, while SFRP4 knockdown effectively suppressed SASP, improved fibroblast proliferation, and enhanced ECM integrity, suggesting its potential as a target for anti-aging interventions (Takaya et al., 2022). A study found that UVB-induced fibroblast senescence is mediated by p16INK4a activation, with Allomyrina dichotoma larvae extract reducing ROS accumulation, suppressing MMP-1, and restoring COL1A1, ultimately preventing ECM degradation​ (Kim et al., 2024). In another study, P-MSC-EVs rejuvenate p16INK4a + senescent fibroblasts by delivering miR-145-5p, activating Erk/Akt signaling, and enhancing tissue regeneration (Su et al., 2023).

The NF-κB signaling pathway is another key regulator of fibroblast senescence, particularly through its role in promoting inflammation. NF-κB is a central transcription factor that regulates the matrix-degrading enzymes, expression of pro-inflammatory cytokines, and growth factors (Liu et al., 2017). In senescent fibroblasts, NF-κB signaling is persistently activated in response to DNA damage, oxidative stress, and other stimuli. Once activated, NF-κB translocates to the nucleus and promotes the expression of pro-inflammatory cytokines such as IL-6, TNF-α, and IL-1β, which are key components of the SASP. The cytokines and MMPs released from senescent fibroblasts contribute to a pro-inflammatory microenvironment and ECM degradation, which accelerates skin aging. This inflammatory feedback loop, driven by NF-κB, further amplifies the senescence phenotype and promotes skin aging (Salminen et al., 2012; Nelson et al., 2018; Chien et al., 2011). Trentini et al. investigated apple-derived nanovesicles (ADNVs) as potential anti-aging agents, revealing that ADNVs enhance collagen synthesis while reducing MMP-1, MMP-8, and MMP-9 expression through the downregulation of Toll-like receptor 4 (TLR4)-NF-κB signaling, highlighting their role in modulating fibroblast senescence and ECM integrity (Trentini et al., 2022). Meanwhile, Li et al. explored the role of SIRT7 in skin immune function and inflammation, showing that SIRT7 downregulation with age reduces TLR2-mediated NF-κB activation, thereby dampening pro-inflammatory responses in dermal fibroblasts, a mechanism potentially linked to decreased skin reactivity in aged individuals ​ (Li et al., 2022). Further emphasizing NF-κB’s role, Harada et al. found that constitutive activation of IKKβ (a key regulator of NF-κB) prevents stress-induced fibroblast senescence by sustaining Ezh2 expression, suppressing p16INK4a activation, and counteracting SASP-associated inflammation (Harada et al., 2024). Woo et al. showed that leaf extract from Isatis tinctoria L. prevents fibroblast senescence through inhibition of the mTOR/NF-κB signaling pathway, thereby reducing SASP secretion and inflammation (Woo et al., 2022). Additionally, Kim et al. identified a novel Morus alba-derived compound (GDHBA) that mitigates TNF-α-induced oxidative damage and inflammation in human dermal fibroblasts, significantly reducing MMP-1 levels via NF-κB and MAPK/AP-1 inhibition (Kim et al., 2022).

**FIGURE 3:**
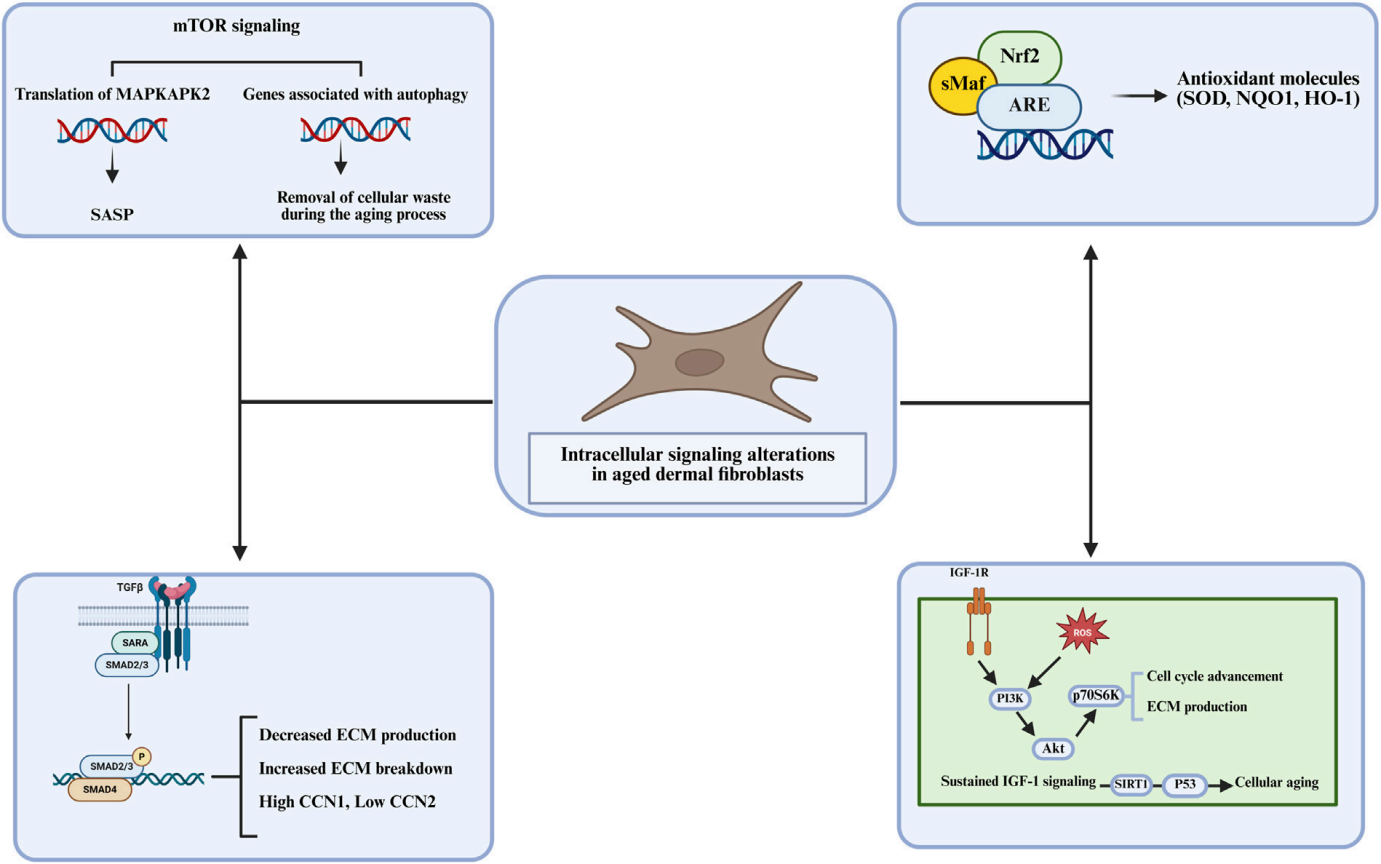
The intracellular signaling alterations in aged dermal fibroblasts, focusing on key pathways that influence cellular aging including, ECM production, autophagy, and antioxidant molecule regulation.

The mTOR signaling pathway, particularly through mTORC1, is a key regulator of cellular growth and metabolism. In dermal fibroblasts, mTORC1 coordinates cellular responses to nutrient availability, stress, and growth factors. Under normal conditions, mTORC1 regulates protein synthesis and inhibits autophagy (Figure 3) (Ben-Sahra and Manning, 2017; Panwar et al., 2023). However, in senescent fibroblasts, mTORC1 activation leads to the autophagy suppression, which is important for maintaining cellular homeostasis by degrading damaged proteins and organelles (Carroll et al., 2017). In the absence of efficient autophagy, cellular damage accumulates, contributing to senescence. Furthermore, mTORC1 activation promotes the expression of SASP factors, which exacerbate the inflammatory environment in aged skin (Kang and Elledge, 2016; Young and Narita, 2010). The mTOR pathway is thus a critical regulator of both the cellular maintenance processes and the inflammatory responses that drive skin aging. Li et al. identified annexin A7 (ANXA7) as a key regulator of senescence-associated heterochromatin foci (SAHF) formation in human dermal fibroblasts via the AMPK/mTOR pathway, showing that inhibiting ANXA7 with ABO enhances mTOR activation while suppressing AMPK phosphorylation, which leads to reduced SAHF formation and altered chromatin remodeling (Li N. et al., 2023).

In addition to these pathways, NRF2 (Nuclear factor erythroid 2-related factor 2) serves as a key regulator in protecting fibroblasts from oxidative stress. NRF2 regulates the expression of antioxidant enzymes that counteract oxidative damage, a major contributor to fibroblast senescence (Kim et al., 2010; Ngo and Duennwald, 2022). As we age, NRF2 activity declines, leading to increased oxidative stress that further exacerbates DNA damage, ECM degradation, and the onset of senescence. Enhancing NRF2 activity has been proposed as a strategy to reduce oxidative damage and delay the progression of skin aging (Medoro et al., 2024). Fang et al. reported that activation of NRF2 by Poria cocos extract shields fibroblasts from H_2_O_2_-induced oxidative damage by enhancing antioxidant responses and suppressing TGF-β/Smad7 signaling, which otherwise drives collagen degradation and ECM instability (Fang et al., 2021). Similarly, Liu et al. found that Urolithin A (UroA) activates NRF2/ARE signaling and mitophagy via the SIRT3-FOXO3-PINK1-PARKIN pathway, effectively reducing ROS accumulation, limiting SASP expression, and preventing fibroblast senescence (Liu et al., 2022). In addition, Takaya et al. observed that Cistanche deserticola polysaccharides enhance NRF2/HO-1 signaling, leading to decreased ROS levels, suppressed SASP markers, and improved fibroblast proliferation, ultimately delaying oxidative damage-induced skin aging (Takaya et al., 2023). In line with this, Lee et al. identified Galangin, a flavonoid, as an NRF2 activator that counteracts H_2_O_2_-induced fibroblast senescence by stimulating SIRT1-PGC-1α/Nrf2 signaling, increasing antioxidant enzyme expression, and maintaining ECM integrity (Lee et al., 2022). NRF2’s role extends to protecting fibroblasts from glucocorticoid-induced skin aging (Kang et al., 2024). Additionally, Darawsha et al. emphasized the synergistic effects of carotenoids, polyphenols, and estradiol in NRF2-mediated mitochondrial protection, which help to maintain collagen integrity and suppress MMP-1 levels, counteracting fibroblast senescence (Darawsha et al., 2024).

The TGF-β signaling pathway is another key mediator of fibroblast senescence, particularly through its role in fibrosis and ECM remodeling. TGF-β is a potent regulator of fibroblast function, and its signaling through the SMAD proteins promotes the expression of ECM components such as collagen. In senescent fibroblasts, TGF-β signaling is upregulated, leading to excessive ECM deposition and fibrosis, which results in formation of wrinkles and stiffening of the skin (Yu et al., 2021). Chronic TGF-β signaling also contributes to the pro-inflammatory environment that drives senescence and aging in dermal tissues. Park et al. highlighted leucine-rich alpha-2-glycoprotein 1 as a key factor in maintaining ECM integrity through TGF-β activation, enhancing collagen production while suppressing MMP-1 expression​ (Park et al., 2023). The developmental stage-specific interactions between TGFBR2 and DNMT3A suggest an epigenetic regulation of fibroblast aging, further influencing ECM remodeling (Tomela et al., 2021). Human ceramides have also been demonstrated to promote collagen and fibrillin synthesis via TGF-β and FGF2 signaling, improving skin elasticity and potentially counteracting structural decline (Sugahara et al., 2022). Santamarine protects dermal fibroblasts from UVA-induced photoaging by suppressing MAPK/AP-1 and activating TGF-β/Smad, reducing MMP-1 expression and restoring collagen production (Oh et al., 2021). Additionally, the anti-aging potential of *Acheta domesticus* extract has been explored, demonstrating the ability to stimulate TGF-β1 expression, inhibit collagenase, and prevent ECM degradation (Yeerong et al., 2024). Research on the effects of cortisol on collagen homeostasis has shown that cortisol suppresses type I collagen production through glucocorticoid receptor signaling. Furthermore, AP collagen peptides have been found to prevent this inhibition by blocking glucocorticoid receptor activation and restoring TGF-β signaling, providing a potential strategy to counteract stress-induced skin aging (Chae et al., 2021).

Finally, IGF-1 signaling promotes fibroblast proliferation and ECM synthesis. IGF-1 activates the PI3K/Akt pathway, which supports cell survival, growth, and collagen production (Khan et al., 2025). As IGF-1 signaling declines with age, fibroblasts lose their proliferative capacity and their ability to maintain the integrity of the ECM, leading to thinning of the dermis and decreased skin elasticity. This reduction in IGF-1 signaling is strongly related to the aging process and the reduced regenerative capacity of fibroblasts in aging skin (Zhang J. et al., 2024). Xu et al. investigated oleanolic acid as an anti-aging agent, demonstrating that it modulates IGF-1 signaling and downregulates PI3K/AKT/mTOR activity, leading to reduced SASP cytokines and improved fibroblast function (Xu et al., 2025). Similarly, Wen et al. studied the galangin’s protective properties in H_2_O_2_-induced oxidative stress in dermal fibroblasts, showing that it activates IGF-1R signaling to enhance collagen synthesis and inhibit inflammation (Wen et al., 2020). Further emphasizing IGF-1’s role in fibroblast health, Mahajan et al. examined creatine and nicotinamide, highlighting their antioxidant properties and ability to prevent fibroblast senescence by maintaining IGF-1 expression (Mahajan et al., 2021). Lee et al. extended this work by demonstrating that IGF-1 increases biglycan and decorin synthesis, which stabilizes collagen and protects against ECM degradation (Lee et al., 2021). Echinacoside promotes collagen synthesis and fibroblast survival by activating IGF-1/IGF-1R pathways, counteracting UVB-induced photoaging and oxidative damage (Wen et al., 2025). Along with that, the loss of IGF-1 expression in aged fibroblasts has been linked to impaired keratinocyte responses to UVB radiation, leading to increased photocarcinogenesis, whereas fractionated laser resurfacing has been shown to restore IGF-1 levels and improve skin resilience (Spandau et al., 2021).

## 5 Future direction

Research on fibroblast senescence and skin aging has rapidly evolved, with novel therapeutic strategies emerging to mitigate cellular dysfunction, chronic inflammation, and ECM degradation. The future of fibroblast senescence research will focus on targeted senotherapeutics, stem cell-based interventions, metabolic and epigenetic reprogramming, and biomaterial applications, all of which hold promise for delaying or reversing age-related skin deterioration. One of the most promising avenues for future research is the development of senolytics and senomorphics to selectively eliminate senescent fibroblasts or suppress the SASP. Recent studies have identified small molecules, such as ABT-263 (Navitoclax) and Quercetin, that target senescent fibroblasts by modulating the Bcl-2 family of proteins, thereby reducing inflammation and restoring tissue homeostasis (Widgerow et al., 2024). Additionally, nutraceuticals such as resveratrol and fisetin have shown potential in reducing oxidative stress and inhibiting SASP-associated cytokines like IL-6, IL-8, and MMPs (Wyles et al., 2024). Future clinical studies should focus on optimizing the safety and efficacy of these compounds for dermatological applications. Stem cell-based approaches, particularly MSC-EVs, offer a promising strategy for fibroblast rejuvenation. MSC-EVs have been shown to transfer bioactive molecules, such as miRNAs and proteins, that suppress fibroblast senescence, enhance collagen synthesis, and modulate inflammatory responses (Liu J. et al., 2024). However, standardizing EV isolation and characterization protocols, as well as optimizing delivery methods, remain major challenges. Future studies should aim to enhance the specificity and longevity of EV-based fibroblast therapies for clinical applications. In addition, metabolic dysregulation has an important role in fibroblast senescence, with studies indicating that senescent fibroblasts undergo metabolic shifts, including mitochondrial dysfunction and increased glycolysis. Caloric restriction mimetics, such as spermidine and nicotinamide riboside, undergo investigation for their potential to restore mitochondrial homeostasis and extend the fibroblast lifespan (Wiley and Campisi, 2021). Moreover, epigenetic alterations, including histone acetylation and DNA methylation, regulate fibroblast senescence, making epigenetic drugs such as histone deacetylase (HDAC) inhibitors potential candidates for reversing fibroblast aging (Wang K. et al., 2022). Recent findings suggest that the skin microbiome is crucial in modulating fibroblast function and inflammation. Dysbiosis, characterized by an imbalance in microbial communities, has been linked to chronic low-grade inflammation and increased oxidative stress in dermal fibroblasts. Probiotic and postbiotic formulations are being explored as potential interventions to modulate fibroblast aging and improve skin resilience (Ratanapokasatit et al., 2022). Future studies should aim to identify particular bacterial strains and microbial metabolites capable of promoting fibroblast longevity. Innovative biomaterials are being developed to support fibroblast function, accelerate wound healing, and combat skin aging. Hydrogel-based scaffolds enriched with growth factors, such as PDGF and epidermal growth factor, have demonstrated efficacy in promoting fibroblast proliferation and collagen synthesis (Sindhi et al., 2025; Chen et al., 2025). Additionally, bioengineered matrices that mimic the native ECM environment may serve as platforms for fibroblast transplantation and skin regeneration therapies. Future research should focus on optimizing biomaterial properties to enhance fibroblast survival and functional integration (Xing et al., 2020).

## 6 Conclusion

The process of dermal aging is closely linked to the functional decline and senescence of fibroblasts, which disrupts extracellular matrix homeostasis, reduces skin elasticity, and promotes chronic inflammation. The interplay between cellular stressors, mitochondrial dysfunction, and epigenetic alterations contributes to fibroblast senescence and the secretion of the senescence-associated secretory phenotype (SASP), further exacerbating skin degeneration. Emerging evidence suggests that targeting fibroblast senescence through senolytics, stem cell-derived extracellular vesicles, and metabolic interventions holds promise for reversing or mitigating age-related skin deterioration. While significant progress has been made in understanding fibroblast aging, future research should focus on optimizing therapeutic strategies to enhance fibroblast longevity, restore ECM integrity, and improve overall skin health. Advances in regenerative medicine, biomaterials, and molecular interventions may provide new avenues for delaying or reversing dermal aging, ultimately improving skin function and resilience against environmental stressors.

## References

[B1] Acosta J. C. O'Loghlen A. Banito A. Guijarro M. V. Augert A. Raguz S. (2008). Chemokine signaling via the CXCR2 receptor reinforces senescence. Cell 133 (6), 1006–1018. 10.1016/j.cell.2008.03.038 18555777

[B2] Adamus J. Aho S. Meldrum H. Bosko C. Lee J. M. (2014). p16INK4A influences the aging phenotype in the living skin equivalent. J. Invest Dermatol 134 (4), 1131–1133. 10.1038/jid.2013.468 24335897 PMC3961602

[B3] Asharaf S. Chakraborty K. Paulose S. K. Dhara S. Chakraborty R. D. Varghese C. (2024). Photoprotective sulfated mannogalactan from heterotrophic Bacillus velezensis blocks UV-A mediated matrix metalloproteinase expression and nuclear DNA damage in human dermal fibroblast. J. Photochem Photobiol. B 260, 113022. 10.1016/j.jphotobiol.2024.113022 39288553

[B4] Bashir M. M. Sharma M. R. Werth V. P. (2009). TNF-α production in the skin. Archives Dermatological Res. 301 (1), 87–91. 10.1007/s00403-008-0893-7 18825399

[B5] Ben-Sahra I. Manning B. D. (2017). mTORC1 signaling and the metabolic control of cell growth. Curr. Opin. Cell Biol. 45, 72–82. 10.1016/j.ceb.2017.02.012 28411448 PMC5545101

[B6] Bian X. Li B. Tang H. Li Q. Hu W. Wei Q. (2022). Extracellular vesicles derived from fibroblasts induced with or without high glucose exert opposite effects on wound healing and angiogenesis. Front. Surg. 9, 1065172. 10.3389/fsurg.2022.1065172 36518227 PMC9742241

[B7] Blokland K. E. C. Pouwels S. D. Schuliga M. Knight D. A. Burgess J. K. (2020). Regulation of cellular senescence by extracellular matrix during chronic fibrotic diseases. Clin. Sci. 134 (20), 2681–2706. 10.1042/CS20190893 PMC7578566 33084883

[B8] Bogdanowicz P. Roullet N. Bensadoun P. Bessou-Touya S. Lemaitre J. M. Duplan H. (2023). Reduction of senescence-associated secretory phenotype and exosome-shuttled miRNAs by Haritaki fruit extract in senescent dermal fibroblasts. Int. J. Cosmet. Sci. 45 (4), 488–499. 10.1111/ics.12858 36940283

[B9] Boraldi F. Lofaro F. D. Bonacorsi S. Mazzilli A. Garcia-Fernandez M. Quaglino D. (2024). The role of fibroblasts in skin homeostasis and repair. Biomedicines 12 (7), 1586. 10.3390/biomedicines12071586 39062158 PMC11274439

[B10] Boxman I. L. A. Ruwhof C. Boerman O. C. Löwik C. W. Ponec M. (1996). Role of fibroblasts in the regulation of proinflammatory interleukin IL-1, IL-6 and IL-8 levels induced by keratinocyte-derived IL-1. Archives Dermatological Res. 288 (7), 391–398. 10.1007/BF02507108 8818187

[B11] Carroll B. Nelson G. Rabanal-Ruiz Y. Kucheryavenko O. Dunhill-Turner N. A. Chesterman C. C. (2017). Persistent mTORC1 signaling in cell senescence results from defects in amino acid and growth factor sensing. J. Cell Biol. 216 (7), 1949–1957. 10.1083/jcb.201610113 28566325 PMC5496614

[B12] Chae M. Bae I. H. Lim S. H. Jung K. Roh J. Kim W. (2021). AP collagen peptides prevent cortisol-induced decrease of collagen type I in human dermal fibroblasts. Int. J. Mol. Sci. 22 (9), 4788. 10.3390/ijms22094788 33946465 PMC8125628

[B13] Chambers E. S. Vukmanovic-Stejic M. Shih B. B. Trahair H. Subramanian P. Devine O. P. (2021). Recruitment of inflammatory monocytes by senescent fibroblasts inhibits antigen-specific tissue immunity during human aging. Nat. Aging 1 (1), 101–113. 10.1038/s43587-020-00010-6 37118005

[B14] Charoensin S. Weera W. (2022). Preventive effect of nuciferine on H(2)O(2)-induced fibroblast senescence and pro-inflammatory cytokine gene expression. Molecules 27 (23), 8148. 10.3390/molecules27238148 36500241 PMC9741010

[B15] Chen L. Xiao L. Ma Y. Xie P. Liu J. Wang C. (2025). Bioengineered composite hydrogel scaffold for accelerated skin regeneration and wound repair. Chem. Eng. J. 504, 158773. 10.1016/j.cej.2024.158773

[B16] Chen Z. Xu X. Lu Y. (2024). Editorial: epithelial immune microenvironment and inflammatory skin diseases. Front. Immunol. 15, 1428209. 10.3389/fimmu.2024.1428209 38831926 PMC11144847

[B17] Chien Y. Scuoppo C. Wang X. Fang X. Balgley B. Bolden J. E. (2011). Control of the senescence-associated secretory phenotype by NF-κB promotes senescence and enhances chemosensitivity. Genes Dev. 25 (20), 2125–2136. 10.1101/gad.17276711 21979375 PMC3205583

[B18] Chin T. Lee X. E. Ng P. Y. Lee Y. Dreesen O. (2023). The role of cellular senescence in skin aging and age-related skin pathologies. Front. Physiol. 14, 1297637. 10.3389/fphys.2023.1297637 38074322 PMC10703490

[B19] Choi E. J. Kil I. S. Cho E. G. (2020). Extracellular vesicles derived from senescent fibroblasts attenuate the dermal effect on keratinocyte differentiation. Int. J. Mol. Sci. 21 (3), 1022. 10.3390/ijms21031022 32033114 PMC7037765

[B20] Cole M. A. Quan T. Voorhees J. J. Fisher G. J. (2018). Extracellular matrix regulation of fibroblast function: redefining our perspective on skin aging. J. Cell Commun. Signal. 12 (1), 35–43. 10.1007/s12079-018-0459-1 29455303 PMC5842211

[B21] Coppé J. P. Desprez P. Y. Krtolica A. Campisi J. (2010). The senescence-associated secretory phenotype: the dark side of tumor suppression. Annu. Rev. Pathol. 5, 99–118. 10.1146/annurev-pathol-121808-102144 20078217 PMC4166495

[B22] Darawsha A. Trachtenberg A. Sharoni Y. (2024). ARE/Nrf2 transcription system involved in carotenoid, polyphenol, and estradiol protection from rotenone-induced mitochondrial oxidative stress in dermal fibroblasts. Antioxidants (Basel) 13 (8), 1019. 10.3390/antiox13081019 39199263 PMC11351643

[B23] Di X. Gao X. Peng L. Ai J. Jin X. Qi S. (2023). Cellular mechanotransduction in health and diseases: from molecular mechanism to therapeutic targets. Signal Transduct. Target. Ther. 8 (1), 282. 10.1038/s41392-023-01501-9 37518181 PMC10387486

[B24] Dong C. Lin J. M. Lu X. Zhu J. Lin L. Xu J. (2024). Fibroblasts with high matrix metalloproteinase 2 expression regulate CD8+ T-cell residency and inflammation via CD100 in psoriasis. Br. J. Dermatology 191 (3), 405–418. 10.1093/bjd/ljae205 38752329

[B25] Esterly A. T. Zapata H. J. (2024). The quest to define senescence. Front. Genet. 15, 1396535. 10.3389/fgene.2024.1396535 38660674 PMC11039885

[B26] Ezure T. Sugahara M. Amano S. (2019). Senescent dermal fibroblasts negatively influence fibroblast extracellular matrix-related gene expression partly via secretion of complement factor D. BioFactors 45 (4), 556–562. 10.1002/biof.1512 31026383 PMC6850482

[B27] Fang C. L. Paul C. R. Day C. H. Chang R. L. Kuo C. H. Ho T. J. (2021). Poria cocos (Fuling) targets TGFβ/Smad7 associated collagen accumulation and enhances Nrf2-antioxidant mechanism to exert anti-skin aging effects in human dermal fibroblasts. Environ. Toxicol. 36 (5), 729–736. 10.1002/tox.23075 33336893

[B28] Fang X. Zhang S. Wu M. Luo Y. Chen X. Zhou Y. (2024). Systemic comparison of molecular characteristics in different skin fibroblast senescent models. Chin. Med. J. 10.1097/CM9.0000000000003312 PMC12407169 39329281

[B29] Farage M. A. Miller K. W. Elsner P. Maibach H. I. (2008). Intrinsic and extrinsic factors in skin ageing: a review. Int. J. Cosmet. Sci. 30 (2), 87–95. 10.1111/j.1468-2494.2007.00415.x 18377617

[B30] Faria A. V. S. Andrade S. S. (2024). Decoding the impact of ageing and environment stressors on skin cell communication. Biogerontology 26 (1), 3. 10.1007/s10522-024-10145-3 39470857

[B31] Feng C. Chen X. Yin X. Jiang Y. Zhao C. (2024). Matrix metalloproteinases on skin photoaging. J. Cosmet. Dermatol 23 (12), 3847–3862. 10.1111/jocd.16558 39230065 PMC11626319

[B32] Fisher G. J. Shao Y. He T. Qin Z. Perry D. Voorhees J. J. (2016). Reduction of fibroblast size/mechanical force down-regulates TGF-β type II receptor: implications for human skin aging. Aging Cell 15 (1), 67–76. 10.1111/acel.12410 26780887 PMC4717276

[B33] Fisher G. J. Wang B. Cui Y. Shi M. Zhao Y. Quan T. (2023). Skin aging from the perspective of dermal fibroblasts: the interplay between the adaptation to the extracellular matrix microenvironment and cell autonomous processes. J. Cell Commun. Signal. 17 (3), 523–529. 10.1007/s12079-023-00743-0 37067763 PMC10409944

[B34] Fligiel S. E. Varani J. Datta S. C. Kang S. Fisher G. J. Voorhees J. J. (2003). Collagen degradation in aged/photodamaged skin *in vivo* and after exposure to matrix metalloproteinase-1 *in vitro* . J. Invest Dermatol 120 (5), 842–848. 10.1046/j.1523-1747.2003.12148.x 12713591

[B35] Franco A. C. Martini H. Victorelli S. Lagnado A. B. Wyles S. P. Rowsey J. L. (2025). Senescent cell transplantation into the skin induces age-related peripheral dysfunction and cognitive decline. Aging Cell 24 (1), e14340. 10.1111/acel.14340 39374134 PMC11709089

[B36] Frediani E. Anceschi C. Ruzzolini J. Ristori S. Nerini A. Laurenzana A. (2024). Divergent regulation of long non-coding RNAs H19 and PURPL affects cell senescence in human dermal fibroblasts. Geroscience 47, 2079–2097. 10.1007/s11357-024-01399-3 39438391 PMC11979041

[B37] Freitas-Rodríguez S. Folgueras A. R. López-Otín C. (2017). The role of matrix metalloproteinases in aging: tissue remodeling and beyond. Biochimica Biophysica Acta (BBA) - Mol. Cell Res. 1864 (11, Part A), 2015–2025. 10.1016/j.bbamcr.2017.05.007 28499917

[B38] Fulop T. Larbi A. Pawelec G. Khalil A. Cohen A. A. Hirokawa K. (2023). Immunology of aging: the birth of inflammaging. Clin. Rev. Allergy Immunol. 64 (2), 109–122. 10.1007/s12016-021-08899-6 34536213 PMC8449217

[B39] Gilaberte Y. Prieto-Torres L. Pastushenko I. Juarranz Á. (2016). “Chapter 1 - anatomy and function of the skin,” in Nanoscience in dermatology. Editors Hamblin M. R. Avci P. Prow T. W. (Boston: Academic Press), 1–14.

[B40] Gruber J. V. Ludwig P. Holtz R. (2013). Modulation of cellular senescence in fibroblasts and dermal papillae cells *in vitro* . J. Cosmet. Sci. 64 (2), 79–87. 23578831

[B41] Guo J. Huang X. Dou L. Yan M. Shen T. Tang W. (2022). Aging and aging-related diseases: from molecular mechanisms to interventions and treatments. Signal Transduct. Target. Ther. 7 (1), 391. 10.1038/s41392-022-01251-0 36522308 PMC9755275

[B42] Haj M. Levon A. Frey Y. Hourvitz N. Campisi J. Tzfati Y. (2023). Accelerated replicative senescence of ataxia-telangiectasia skin fibroblasts is retained at physiologic oxygen levels, with unique and common transcriptional patterns. Aging Cell 22 (8), e13869. 10.1111/acel.13869 37254625 PMC10410012

[B43] Harada M. Su-Harada K. Kimura T. Ono K. Ashida N. (2024). Sustained activation of NF-κB through constitutively active IKKβ leads to senescence bypass in murine dermal fibroblasts. Cell Cycle 23 (3), 308–327. 10.1080/15384101.2024.2325802 38461418 PMC11057680

[B44] Horn G. Schäfers C. Thiermann H. Völkl S. Schmidt A. Rothmiller S. (2022). Sulfur mustard single-dose exposure triggers senescence in primary human dermal fibroblasts. Archives Toxicol. 96 (11), 3053–3066. 10.1007/s00204-022-03346-7 PMC9525386 35906424

[B45] Hovest M. G. Brüggenolte N. Hosseini K. S. Krieg T. Herrmann G. (2006). Senescence of human fibroblasts after psoralen photoactivation is mediated by ATR kinase and persistent DNA damage foci at telomeres. Mol. Biol. Cell 17 (4), 1758–1767. 10.1091/mbc.e05-08-0701 16436511 PMC1415309

[B46] Huang R.-X. Zhou P.-K. (2020). DNA damage response signaling pathways and targets for radiotherapy sensitization in cancer. Signal Transduct. Target. Ther. 5 (1), 60. 10.1038/s41392-020-0150-x 32355263 PMC7192953

[B47] Hussein R. S. Bin Dayel S. Abahussein O. El-Sherbiny A. A. (2025). Influences on skin and intrinsic aging: biological, environmental, and therapeutic insights. J. Cosmet. Dermatology 24 (2), e16688. 10.1111/jocd.16688 PMC11845971 39604792

[B48] Imb M. Véghelyi Z. Maurer M. Kühnel H. (2024). Exploring senolytic and senomorphic properties of medicinal plants for anti-aging therapies. Int. J. Mol. Sci. 25 (19), 10419. 10.3390/ijms251910419 39408750 PMC11476546

[B49] Jin P. Feng X. Huang C. Li J. Wang H. Wang X. (2024). Oxidative stress and cellular senescence: roles in tumor progression and therapeutic opportunities. MedComm – Oncol. 3 (4), e70007. 10.1002/mog2.70007

[B50] Johnson B. Z. Stevenson A. W. Prêle C. M. Fear M. W. Wood F. M. (2020). The role of IL-6 in skin fibrosis and cutaneous wound healing. Biomedicines 8 (5), 101. 10.3390/biomedicines8050101 32365896 PMC7277690

[B51] Junker H. (2024). Multiscale mechanics of skin. Zurich, Netherlands: ETH Zurich.

[B52] Kabashima K. Honda T. Ginhoux F. Egawa G. (2019). The immunological anatomy of the skin. Nat. Rev. Immunol. 19 (1), 19–30. 10.1038/s41577-018-0084-5 30429578

[B53] Kang C. Elledge S. J. (2016). How autophagy both activates and inhibits cellular senescence. Autophagy 12 (5), 898–899. 10.1080/15548627.2015.1121361 27129029 PMC4854549

[B54] Kang J. Chen W. Xia J. Li Y. Yang B. Chen B. (2008). Extracellular matrix secreted by senescent fibroblasts induced by UVB promotes cell proliferation in HaCaT cells through PI3K/AKT and ERK signaling pathways. Int. J. Mol. Med. 21 (6), 777–784. 18506372

[B55] Kang W. Ha Y. Jung Y. Lee H. Park T. (2024). Nerol mitigates dexamethasone-induced skin aging by activating the Nrf2 signaling pathway in human dermal fibroblasts. Life Sci. 356, 123034. 10.1016/j.lfs.2024.123034 39236900

[B56] Khan M. Z. Zugaza J. L. Torres Aleman I. (2025). The signaling landscape of insulin-like growth factor 1. J. Biol. Chem. 301 (1), 108047. 10.1016/j.jbc.2024.108047 39638246 PMC11748690

[B57] Khavinson V. Linkova N. Dyatlova A. Kantemirova R. Kozlov K. (2022). Senescence-associated secretory phenotype of cardiovascular system cells and inflammaging: perspectives of peptide regulation. Cells 12 (1), 106. 10.3390/cells12010106 36611900 PMC9818427

[B58] Kim J. Cha Y.-N. Surh Y.-J. (2010). A protective role of nuclear factor-erythroid 2-related factor-2 (Nrf2) in inflammatory disorders. Mutat. Research/Fundamental Mol. Mech. Mutagen. 690 (1), 12–23. 10.1016/j.mrfmmm.2009.09.007 19799917

[B59] Kim K. Kim C. E. Baek D. J. Park E. Y. Oh Y. S. (2024). Prevention of UVB-induced photoaging by an ethyl acetate fraction from Allomyrina dichotoma larvae and its potential mechanisms in human dermal fibroblasts. Int. J. Mol. Sci. 25 (14), 7850. 10.3390/ijms25147850 39063091 PMC11277254

[B60] Kim K. S. Choi Y. J. Jang D. S. Lee S. (2022). 2-O-β-d-Glucopyranosyl-4,6-dihydroxybenzaldehyde isolated from Morus alba (mulberry) fruits suppresses damage by regulating oxidative and inflammatory responses in TNF-α-induced human dermal fibroblasts. Int. J. Mol. Sci. 23 (23), 14802. 10.3390/ijms232314802 36499128 PMC9735759

[B61] Kita A. Yamamoto S. Saito Y. Chikenji T. S. (2024). Cellular senescence and wound healing in aged and diabetic skin. Front. Physiol. 15, 1344116. 10.3389/fphys.2024.1344116 38440347 PMC10909996

[B62] Koczkowska M. Kostecka A. Zawrzykraj M. Myszczyński K. Skoniecka A. Deptuła M. (2025). Identifying differentiation markers between dermal fibroblasts and adipose-derived mesenchymal stromal cells (AD-MSCs) in human visceral and subcutaneous tissues using single-cell transcriptomics. Stem Cell Res. & Ther. 16 (1), 64. 10.1186/s13287-025-04185-w 39934849 PMC11818286

[B63] Kolář M. Szabo P. Dvořánková B. Lacina L. Gabius H. J. Strnad H. (2012). Upregulation of IL-6, IL-8 and CXCL-1 production in dermal fibroblasts by normal/malignant epithelial cells *in vitro*: immunohistochemical and transcriptomic analyses. Biol. Cell 104 (12), 738–751. 10.1111/boc.201200018 23043537

[B64] Konstantinou E. Longange E. Kaya G. (2024). Mechanisms of senescence and anti-senescence strategies in the skin. Biol. (Basel) 13 (9), 647. 10.3390/biology13090647 PMC11428750 39336075

[B65] Koskela von Sydow A. Janbaz C. Kardeby C. Repsilber D. Ivarsson M. (2016). IL-1α counteract TGF-β regulated genes and pathways in human fibroblasts. J. Cell Biochem. 117 (7), 1622–1632. 10.1002/jcb.25455 26629874

[B66] Kudlova N. De Sanctis J. B. Hajduch M. (2022). Cellular senescence: molecular targets, biomarkers, and senolytic drugs. Int. J. Mol. Sci. 23 (8), 4168. 10.3390/ijms23084168 35456986 PMC9028163

[B67] Kühnel H. Seiler M. Feldhofer B. Ebrahimian A. Maurer M. (2025). Ganoderma lucidum extract modulates gene expression profiles associated with antioxidant defense, cytoprotection, and senescence in human dermal fibroblasts: investigation of quantitative gene expression by qPCR. Curr. Issues Mol. Biol. 47 (2), 130. 10.3390/cimb47020130 39996851 PMC11854148

[B68] Lee H. Lim J. Oh J. H. Cho S. Chung J. H. (2021). IGF-1 upregulates biglycan and decorin by increasing translation and reducing ADAMTS5 expression. Int. J. Mol. Sci. 22 (3), 1403. 10.3390/ijms22031403 33573338 PMC7866853

[B69] Lee H. J. Kim M. (2022). Skin barrier function and the microbiome. Int. J. Mol. Sci. 23 (21), 13071. 10.3390/ijms232113071 36361857 PMC9654002

[B70] Lee J. J. Ng S. C. Hsu J. Y. Liu H. Chen C. J. Huang C. Y. (2022). Galangin reverses H(2)O(2)-induced dermal fibroblast senescence via SIRT1-PGC-1α/nrf2 signaling. Int. J. Mol. Sci. 23 (3), 1387. 10.3390/ijms23031387 35163314 PMC8836071

[B71] Lei W. Jia L. Wang Z. Liang Z. Zhao A. Liu Y. (2023). CC chemokines family in fibrosis and aging: from mechanisms to therapy. Ageing Res. Rev. 87, 101900. 10.1016/j.arr.2023.101900 36871782

[B72] Li G. Tang X. Zhang S. Deng Z. Wang B. Shi W. (2022). Aging-conferred SIRT7 decline inhibits rosacea-like skin inflammation by modulating toll-like receptor 2‒NF-κB signaling. J. Invest Dermatol 142 (10), 2580–2590.e6. 10.1016/j.jid.2022.03.026 35413292

[B73] Li N. Yan X. Cui X. Zhao C. Lin Z. Miao J. (2023). Inhibition of annexin A7 suppresses senescence-associated heterochromatin foci formation and senescence through the AMPK/mTOR pathway in human dermal fibroblasts. J. Cell Biochem. 124 (10), 1603–1614. 10.1002/jcb.30472 37682859

[B74] Li X. Li C. Zhang W. Wang Y. Qian P. Huang H. (2023). Inflammation and aging: signaling pathways and intervention therapies. Signal Transduct. Target. Ther. 8 (1), 239. 10.1038/s41392-023-01502-8 37291105 PMC10248351

[B75] Li Y. Guo M. Li L. Yang F. Xiong L. (2025). Effects of rice fermentation and its bioactive components on UVA-induced oxidative stress and senescence in dermal fibroblasts. Photochem. Photobiol. 101, 392–403. 10.1111/php.14003 39030789

[B76] Liu J. Yu H. Tian Y. Liu N. (2024). “Extracellular vesicles in aging and age-related diseases,” in Extracellular Vesicle: Bbiology and Translational Application. Editor Li Z. (Singapore: Springer), 295–310. 10.1007/978-981-97-5536-3_14

[B77] Liu T. Zhang L. Joo D. Sun S. C. (2017). NF-κB signaling in inflammation. Signal Transduct. Target. Ther. 2 (1), 17023. 10.1038/sigtrans.2017.23 29158945 PMC5661633

[B78] Liu W. Yan F. Xu Z. Chen Q. Ren J. Wang Q. (2022). Urolithin A protects human dermal fibroblasts from UVA-induced photoaging through NRF2 activation and mitophagy. J. Photochem Photobiol. B 232, 112462. 10.1016/j.jphotobiol.2022.112462 35567884

[B79] Liu Y. Li Y. Li N. Teng W. Wang M. Zhang Y. (2016). TGF-β1 promotes scar fibroblasts proliferation and transdifferentiation via up-regulating MicroRNA-21. Sci. Rep. 6, 32231. 10.1038/srep32231 27554193 PMC4995376

[B80] Liu Y. Zhang M. Wang C. Chen H. Su D. Yang C. (2024). Human umbilical cord mesenchymal stromal cell-derived extracellular vesicles induce fetal wound healing features revealed by single-cell RNA sequencing. ACS Nano 18 (21), 13696–13713. 10.1021/acsnano.4c01401 38751164

[B81] Lopes-Paciencia S. Saint-Germain E. Rowell M. C. Ruiz A. F. Kalegari P. Ferbeyre G. (2019). The senescence-associated secretory phenotype and its regulation. Cytokine 117, 15–22. 10.1016/j.cyto.2019.01.013 30776684

[B82] López-Otín C. Blasco M. A. Partridge L. Serrano M. Kroemer G. (2023). Hallmarks of aging: an expanding universe. Cell 186 (2), 243–278. 10.1016/j.cell.2022.11.001 36599349

[B83] Lyamina S. Baranovskii D. Kozhevnikova E. Ivanova T. Kalish S. Sadekov T. (2023). Mesenchymal stromal cells as a driver of inflammaging. Int. J. Mol. Sci. 24 (7), 6372. 10.3390/ijms24076372 37047346 PMC10094085

[B84] Ma W. Zhou S. (2025). Metabolic rewiring in the face of genomic assault: integrating DNA damage response and cellular metabolism. Biomolecules 15 (2), 168. 10.3390/biom15020168 40001471 PMC11852599

[B85] Mahajan A. S. Arikatla V. S. Thyagarajan A. Zhelay T. Sahu R. P. Kemp M. G. (2021). Creatine and nicotinamide prevent oxidant-induced senescence in human fibroblasts. Nutrients 13 (11), 4102. 10.3390/nu13114102 34836359 PMC8622652

[B86] Malaquin N. Martinez A. Rodier F. (2016). Keeping the senescence secretome under control: molecular reins on the senescence-associated secretory phenotype. Exp. Gerontol. 82, 39–49. 10.1016/j.exger.2016.05.010 27235851

[B87] Maret M. Chicheportiche R. Dayer J. M. Gabay C. (2004). Production of intracellular IL-1alpha, IL-1beta, and IL-1Ra isoforms by activated human dermal and synovial fibroblasts: phenotypic differences between human dermal and synovial fibroblasts. Cytokine 25 (5), 193–203. 10.1016/j.cyto.2003.10.003 15036245

[B88] Mavrogonatou E. Angelopoulou M. Rizou S. V. Pratsinis H. Gorgoulis V. G. Kletsas D. (2022). Activation of the JNKs/ATM-p53 axis is indispensable for the cytoprotection of dermal fibroblasts exposed to UVB radiation. Cell Death Dis. 13 (7), 647. 10.1038/s41419-022-05106-y 35879280 PMC9314411

[B89] Mavrogonatou E. Konstantinou A. Kletsas D. (2018). Long-term exposure to TNF-α leads human skin fibroblasts to a p38 MAPK- and ROS-mediated premature senescence. Biogerontology 19 (3), 237–249. 10.1007/s10522-018-9753-9 29582209

[B90] Mavrogonatou E. Papadopoulou A. Pratsinis H. Kletsas D. (2023). Senescence-associated alterations in the extracellular matrix: deciphering their role in the regulation of cellular function. Am. J. Physiology-Cell Physiology 325 (3), C633–C647. 10.1152/ajpcell.00178.2023 37486063

[B91] Medoro A. Saso L. Scapagnini G. Davinelli S. (2024). NRF2 signaling pathway and telomere length in aging and age-related diseases. Mol. Cell Biochem. 479 (10), 2597–2613. 10.1007/s11010-023-04878-x 37917279 PMC11455797

[B92] Mijit M. Caracciolo V. Melillo A. Amicarelli F. Giordano A. (2020). Role of p53 in the regulation of cellular senescence. Biomolecules 10 (3), 420. 10.3390/biom10030420 32182711 PMC7175209

[B93] Narzt M.-S. Pils V. Kremslehner C. Nagelreiter I. M. Schosserer M. Bessonova E. (2021). Epilipidomics of senescent dermal fibroblasts identify lysophosphatidylcholines as pleiotropic senescence-associated secretory phenotype (SASP) factors. J. Investigative Dermatology 141 (4), 993–1006.e15. 10.1016/j.jid.2020.11.020 33333126

[B94] Nelson G. Kucheryavenko O. Wordsworth J. von Zglinicki T. (2018). The senescent bystander effect is caused by ROS-activated NF-κB signalling. Mech. Ageing Dev. 170, 30–36. 10.1016/j.mad.2017.08.005 28837845 PMC5861994

[B95] Ngo V. Duennwald M. L. (2022). Nrf2 and oxidative stress: a general overview of mechanisms and implications in human disease. Antioxidants (Basel) 11 (12), 2345. 10.3390/antiox11122345 36552553 PMC9774434

[B96] Novotná R. Škařupová D. Hanyk J. Ulrichová J. Křen V. Bojarová P. (2023). Hesperidin, hesperetin, rutinose, and rhamnose act as skin anti-aging agents. Molecules 28 (4), 1728. 10.3390/molecules28041728 36838716 PMC9963045

[B97] Ogata Y. Yamada T. Hasegawa S. Sanada A. Iwata Y. Arima M. (2021). SASP-induced macrophage dysfunction may contribute to accelerated senescent fibroblast accumulation in the dermis. Exp. Dermatol 30 (1), 84–91. 10.1111/exd.14205 33010063

[B98] Oh J. H. Kim J. Karadeniz F. Kim H. R. Park S. Y. Seo Y. (2021). Santamarine shows anti-photoaging properties via inhibition of MAPK/AP-1 and stimulation of TGF-β/smad signaling in UVA-irradiated HDFs. Molecules 26 (12), 3585. 10.3390/molecules26123585 34208202 PMC8230857

[B99] Ohgo S. Hasegawa S. Hasebe Y. Mizutani H. Nakata S. Akamatsu H. (2015). Senescent dermal fibroblasts enhance stem cell migration through CCL2/CCR2 axis. Exp. Dermatol 24 (7), 552–554. 10.1111/exd.12701 25808810

[B100] Ohtani N. Yamakoshi K. Takahashi A. Hara E. (2004). The p16INK4a-RB pathway: molecular link between cellular senescence and tumor suppression. J. Med. Invest 51 (3-4), 146–153. 10.2152/jmi.51.146 15460900

[B101] Ohtsuka T. Jensen M. R. Kim H. G. Kim K. T. Lee S. W. (2004). The negative role of cyclin G in ATM-dependent p53 activation. Oncogene 23 (31), 5405–5408. 10.1038/sj.onc.1207693 15077171

[B102] Panwar V. Singh A. Bhatt M. Tonk R. K. Azizov S. Raza A. S. (2023). Multifaceted role of mTOR (mammalian target of rapamycin) signaling pathway in human health and disease. Signal Transduct. Target. Ther. 8 (1), 375. 10.1038/s41392-023-01608-z 37779156 PMC10543444

[B103] Park H. N. Song M. J. Choi Y. E. Lee D. H. Chung J. H. Lee S. T. (2023). LRG1 promotes ECM integrity by activating the TGF-β signaling pathway in fibroblasts. Int. J. Mol. Sci. 24 (15), 12445. 10.3390/ijms241512445 37569820 PMC10418909

[B104] Pereira B. I. Devine O. P. Vukmanovic-Stejic M. Chambers E. S. Subramanian P. Patel N. (2019). Senescent cells evade immune clearance via HLA-E-mediated NK and CD8+ T cell inhibition. Nat. Commun. 10 (1), 2387. 10.1038/s41467-019-10335-5 31160572 PMC6547655

[B105] Pilkington S. M. Bulfone-Paus S. Griffiths C. E. M. Watson R. E. B. (2021). Inflammaging and the skin. J. Investigative Dermatology 141 (4), 1087–1095. 10.1016/j.jid.2020.11.006 33358020

[B106] Plikus M. V. Wang X. Sinha S. Forte E. Thompson S. M. Herzog E. L. (2021). Fibroblasts: origins, definitions, and functions in health and disease. Cell 184 (15), 3852–3872. 10.1016/j.cell.2021.06.024 34297930 PMC8566693

[B107] Pretzsch E. Nieß H. Bösch F. Westphalen C. B. Jacob S. Neumann J. (2022). Age and metastasis – how age influences metastatic spread in cancer. Colorectal cancer as a model. Cancer Epidemiol. 77, 102112. 10.1016/j.canep.2022.102112 35104771

[B108] Promjantuek W. Chaicharoenaudomrung N. Phonchai R. Kunhorm P. Noisa P. (2022). Transgenic immortalization of human dermal fibroblasts mediated through the MicroRNA/SIRT1 pathway. Vivo 36 (1), 140–152. 10.21873/invivo.12685 PMC8765157 34972709

[B109] Qin Z. Balimunkwe R. M. Quan T. (2017). Age-related reduction of dermal fibroblast size upregulates multiple matrix metalloproteinases as observed in aged human skin *in vivo* . Br. J. Dermatol 177 (5), 1337–1348. 10.1111/bjd.15379 28196296 PMC5555832

[B110] Quan T. Little E. Quan H. Qin Z. Voorhees J. J. Fisher G. J. (2013). Elevated matrix metalloproteinases and collagen fragmentation in photodamaged human skin: impact of altered extracellular matrix microenvironment on dermal fibroblast function. J. Invest Dermatol 133 (5), 1362–1366. 10.1038/jid.2012.509 PMC3637921 23466932

[B111] Quan T. Xia W. He T. Calderone K. Bou-Gharios G. Voorhees J. J. (2023). Matrix metalloproteinase-1 expression in fibroblasts accelerates dermal aging and promotes papilloma development in mouse skin. J. Invest Dermatol 143 (9), 1700–1707.e1. 10.1016/j.jid.2023.02.028 36914001 PMC11577279

[B112] Ratanapokasatit Y. Laisuan W. Rattananukrom T. Petchlorlian A. Thaipisuttikul I. Sompornrattanaphan M. (2022). How microbiomes affect skin aging: the updated evidence and current perspectives. Life (Basel) 12 (7), 936. 10.3390/life12070936 35888025 PMC9320090

[B113] Richardson M. (2003). Understanding the structure and function of the skin. Nurs. Times 99 (31), 46–48. 13677123

[B114] Sadangi S. Milosavljevic K. Castro-Perez E. Lares M. Singh M. Altameemi S. (2022). Role of the skin microenvironment in melanomagenesis: epidermal keratinocytes and dermal fibroblasts promote BRAF oncogene-induced senescence escape in melanocytes. Cancers 14 (5), 1233. 10.3390/cancers14051233 35267541 PMC8909265

[B115] Safwan-Zaiter H. Wagner N. Wagner K. D. (2022). P16INK4A-More than a senescence marker. Life (Basel) 12 (9), 1332. 10.3390/life12091332 36143369 PMC9501954

[B116] Salminen A. Kauppinen A. Kaarniranta K. (2012). Emerging role of NF-κB signaling in the induction of senescence-associated secretory phenotype (SASP). Cell. Signal. 24 (4), 835–845. 10.1016/j.cellsig.2011.12.006 22182507

[B117] Serizawa H. (1998). Cyclin-dependent kinase inhibitor p16INK4A inhibits phosphorylation of RNA polymerase II by general transcription factor TFIIH. J. Biol. Chem. 273 (10), 5427–5430. 10.1074/jbc.273.10.5427 9488660

[B118] Shackelford R. E. Innes C. L. Sieber S. O. Heinloth A. N. Leadon S. A. Paules R. S. (2001). The Ataxia telangiectasia gene product is required for oxidative stress-induced G1 and G2 checkpoint function in human fibroblasts. J. Biol. Chem. 276 (24), 21951–21959. 10.1074/jbc.M011303200 11290740

[B119] Shin J. W. Kwon S. H. Choi J. Y. Na J. I. Huh C. H. Choi H. R. (2019). Molecular mechanisms of dermal aging and antiaging approaches. Int. J. Mol. Sci. 20 (9), 2126. 10.3390/ijms20092126 31036793 PMC6540032

[B120] Sierra-Sánchez Á. Kim K. H. Blasco-Morente G. Arias-Santiago S. (2021). Cellular human tissue-engineered skin substitutes investigated for deep and difficult to heal injuries. npj Regen. Med. 6 (1), 35. 10.1038/s41536-021-00144-0 34140525 PMC8211795

[B121] Sindhi K. Pingili R. B. Beldar V. Bhattacharya S. Rahaman J. Mukherjee D. (2025). The role of biomaterials-based scaffolds in advancing skin tissue construct. J. Tissue Viability 34 (2), 100858. 10.1016/j.jtv.2025.100858 39827732

[B122] Smirnova A. Yatsenko E. Baranovskii D. Klabukov I. (2023). Mesenchymal stem cell-derived extracellular vesicles in skin wound healing: the risk of senescent drift induction in secretome-based therapeutics. Mil. Med. Res. 10 (1), 60. 10.1186/s40779-023-00498-0 38031201 PMC10688489

[B123] Smith P. Carroll B. (2024). Senescence in the ageing skin: a new focus on mTORC1 and the lysosome. Febs J. 292, 960–975. 10.1111/febs.17281 39325694 PMC11880983

[B124] Solé-Boldo L. Raddatz G. Schütz S. Mallm J. P. Rippe K. Lonsdorf A. S. (2020). Single-cell transcriptomes of the human skin reveal age-related loss of fibroblast priming. Commun. Biol. 3 (1), 188. 10.1038/s42003-020-0922-4 32327715 PMC7181753

[B125] Song M. J. Kim M. K. Park C. H. Kim H. Lee S. H. Lee D. H. (2024). Downregulation of carnitine acetyltransferase by promoter hypermethylation regulates ultraviolet-induced matrix metalloproteinase-1 expression in human dermal fibroblasts. J. Dermatol Sci. 116 (2), 70–77. 10.1016/j.jdermsci.2024.09.005 39443271

[B126] Song M. J. Park C. H. Kim H. Han S. Lee S. H. Lee D. H. (2023). Carnitine acetyltransferase deficiency mediates mitochondrial dysfunction-induced cellular senescence in dermal fibroblasts. Aging Cell 22 (11), e14000. 10.1111/acel.14000 37828898 PMC10652321

[B127] Song P. An J. Zou M. H. (2020). Immune clearance of senescent cells to combat ageing and chronic diseases. Cells 9 (3), 671. 10.3390/cells9030671 32164335 PMC7140645

[B128] Spandau D. F. Chen R. Wargo J. J. Rohan C. A. Southern D. Zhang A. (2021). Randomized controlled trial of fractionated laser resurfacing on aged skin as prophylaxis against actinic neoplasia. J. Clin. Invest 131 (19), e150972. 10.1172/JCI150972 34428179 PMC8483749

[B129] Srivastava S. (2017). The mitochondrial basis of aging and age-related disorders. Genes (Basel) 8 (12), 398. 10.3390/genes8120398 29257072 PMC5748716

[B130] Su J. Wei Q. Ma K. Wang Y. Hu W. Meng H. (2023). P-MSC-derived extracellular vesicles facilitate diabetic wound healing via miR-145-5p/CDKN1A-mediated functional improvements of high glucose-induced senescent fibroblasts. Burns Trauma 11, tkad010. 10.1093/burnst/tkad010 37860579 PMC10583213

[B131] Sugahara Y. Komorisono M. Kuwajima M. Yoshikawa S. Yoshida S. Maeda K. (2022). Anti-skin-aging effects of human ceramides via collagen and fibrillin expression in dermal fibroblasts. Biosci. Biotechnol. Biochem. 86 (9), 1240–1246. 10.1093/bbb/zbac107 35776962

[B132] Sun Y. (2023). An updated landscape of cellular senescence heterogeneity: mechanisms, technologies and senotherapies. Transl. Med. Aging 7, 46–51.

[B133] Swaroop A. K. Negi P. Kar A. Mariappan E. Natarajan J. Namboori P K K. (2024). Navigating IL-6: from molecular mechanisms to therapeutic breakthroughs. Cytokine & Growth Factor Rev. 76, 48–76. 10.1016/j.cytogfr.2023.12.007 38220583

[B134] Takaya K. Asou T. Kishi K. (2022). Downregulation of senescence-associated secretory phenotype by knockdown of secreted frizzled-related protein 4 contributes to the prevention of skin aging. Aging (Albany NY) 14 (20), 8167–8178. 10.18632/aging.204273 36084952 PMC9648805

[B135] Takaya K. Asou T. Kishi K. (2023). Cistanche deserticola polysaccharide reduces inflammation and aging phenotypes in the dermal fibroblasts through the activation of the NRF2/HO-1 pathway. Int. J. Mol. Sci. 24 (21), 15704. 10.3390/ijms242115704 37958685 PMC10647235

[B136] Tanaka T. Narazaki M. Kishimoto T. (2014). IL-6 in inflammation, immunity, and disease. Cold Spring Harb. Perspect. Biol. 6 (10), a016295. 10.1101/cshperspect.a016295 25190079 PMC4176007

[B137] Tao W. Yu Z. Han J.-D. J. (2024). Single-cell senescence identification reveals senescence heterogeneity, trajectory, and modulators. Cell Metab. 36 (5), 1126–1143.e5. 10.1016/j.cmet.2024.03.009 38604170

[B138] Thulabandu V. Chen D. Atit R. P. (2018). Dermal fibroblast in cutaneous development and healing. Wiley Interdiscip. Rev. Dev. Biol. 7 (2). 10.1002/wdev.307 PMC5814349 29244903

[B139] Tiemann J. Wagner T. Lindenkamp C. Plümers R. Faust I. Knabbe C. (2020). Linking ABCC6 deficiency in primary human dermal fibroblasts of PXE patients to p21-mediated premature cellular senescence and the development of a proinflammatory secretory phenotype. Int. J. Mol. Sci. 21 (24), 9665. 10.3390/ijms21249665 33352936 PMC7766446

[B140] Tivey H. S. Brook A. J. C. Rokicki M. J. Kipling D. Davis T. (2013). p38 (MAPK) stress signalling in replicative senescence in fibroblasts from progeroid and genomic instability syndromes. Biogerontology 14 (1), 47–62. 10.1007/s10522-012-9407-2 23112078 PMC3627027

[B141] Tomela K. Karolak J. A. Ginter-Matuszewska B. Kabza M. Gajecka M. (2021). Influence of TGFBR2, TGFB3, DNMT1, and DNMT3A knockdowns on CTGF, TGFBR2, and DNMT3A in neonatal and adult human dermal fibroblasts cell lines. Curr. Issues Mol. Biol. 43 (1), 276–285. 10.3390/cimb43010023 34204856 PMC8928948

[B142] Torres G. Salladay-Perez I. A. Dhingra A. Covarrubias A. J. (2024). Genetic origins, regulators, and biomarkers of cellular senescence. Trends Genet. 40 (12), 1018–1031. 10.1016/j.tig.2024.08.007 39341687 PMC11717094

[B143] Toutfaire M. Dumortier E. Fattaccioli A. Van Steenbrugge M. Proby C. M. Debacq-Chainiaux F. (2018). Unraveling the interplay between senescent dermal fibroblasts and cutaneous squamous cell carcinoma cell lines at different stages of tumorigenesis. Int. J. Biochem. & Cell Biol. 98, 113–126. 10.1016/j.biocel.2018.03.005 29550586

[B144] Trentini M. Zanolla I. Zanotti F. Tiengo E. Licastro D. Dal Monego S. (2022). Apple derived exosomes improve collagen type I production and decrease MMPs during aging of the skin through downregulation of the NF-κB pathway as mode of action. Cells 11 (24), 3950. 10.3390/cells11243950 36552714 PMC9776931

[B145] Urban L. Čoma M. Lacina L. Szabo P. Sabová J. Urban T. (2023). Heterogeneous response to TGF-β1/3 isoforms in fibroblasts of different origins: implications for wound healing and tumorigenesis. Histochem. Cell Biol. 160 (6), 541–554. 10.1007/s00418-023-02221-5 37707642 PMC10700238

[B146] Uyar B. Palmer D. Kowald A. Murua Escobar H. Barrantes I. Möller S. (2020). Single-cell analyses of aging, inflammation and senescence. Ageing Res. Rev. 64, 101156. 10.1016/j.arr.2020.101156 32949770 PMC7493798

[B147] Vandenberk B. Brouwers B. Hatse S. Wildiers H. (2011). p16INK4a: a central player in cellular senescence and a promising aging biomarker in elderly cancer patients. J. Geriatric Oncol. 2 (4), 259–269. 10.1016/j.jgo.2011.08.004

[B148] Varani J. Dame M. K. Rittie L. Fligiel S. E. G. Kang S. Fisher G. J. (2006). Decreased collagen production in chronologically aged skin: roles of age-dependent alteration in fibroblast function and defective mechanical stimulation. Am. J. Pathology 168 (6), 1861–1868. 10.2353/ajpath.2006.051302 PMC1606623 16723701

[B149] Waldera Lupa D. M. Kalfalah F. Safferling K. Boukamp P. Poschmann G. Volpi E. (2015). Characterization of skin aging-associated secreted proteins (SAASP) produced by dermal fibroblasts isolated from intrinsically aged human skin. J. Invest Dermatol 135 (8), 1954–1968. 10.1038/jid.2015.120 25815425

[B150] Wang B. Han J. Elisseeff J. H. Demaria M. (2024). The senescence-associated secretory phenotype and its physiological and pathological implications. Nat. Rev. Mol. Cell Biol. 25 (12), 958–978. 10.1038/s41580-024-00727-x 38654098

[B151] Wang K. Liu H. Hu Q. Wang L. Liu J. Zheng Z. (2022). Epigenetic regulation of aging: implications for interventions of aging and diseases. Signal Transduct. Target. Ther. 7 (1), 374. 10.1038/s41392-022-01211-8 36336680 PMC9637765

[B152] Wang S. Liang Y. Dai C. (2022). Metabolic regulation of fibroblast activation and proliferation during organ fibrosis. Kidney Dis. (Basel) 8 (2), 115–125. 10.1159/000522417 35527985 PMC9021660

[B153] Weebadda W. K. Jackson T. J. Lin A. W. (2005). Expression of p16INK4A variants in senescent human fibroblasts independent of protein phosphorylation. J. Cell Biochem. 94 (6), 1135–1147. 10.1002/jcb.20372 15668906

[B154] Wen S. Y. Chen J. Y. Chen C. J. Huang C. Y. Kuo W. W. (2020). Protective effects of galangin against H(2) O(2) -induced aging via the IGF-1 signaling pathway in human dermal fibroblasts. Environ. Toxicol. 35 (2), 115–123. 10.1002/tox.22847 31566298

[B155] Wen S. Y. Ng S. C. Chiu Y. T. Tai P. Y. Chen T. J. Chen C. J. (2024). Enhanced SIRT1 activity by galangin mitigates UVB-induced senescence in dermal fibroblasts via p53 acetylation regulation and activation. J. Agric. Food Chem. 72 (42), 23286–23294. 10.1021/acs.jafc.4c05945 39401943

[B156] Wen S. Y. Ng S. C. Noriega L. Chen T. J. Chen C. J. Lee S. D. (2025). Echinacoside promotes collagen synthesis and survival via activation of IGF-1 signaling to alleviate UVB-induced dermal fibroblast photoaging. Biofactors 51 (1), e2152. 10.1002/biof.2152 39780317

[B157] Widgerow A. D. Ziegler M. Garruto J. A. Ionescu L. Shafiq F. Meckfessel M. (2024). Novel strategy for strengthening dermatoporotic skin by managing cellular senescence. J. Drugs Dermatol 23 (9), 748–756. 10.36849/JDD.8388 39231083

[B158] Wiley C. D. Campisi J. (2021). The metabolic roots of senescence: mechanisms and opportunities for intervention. Nat. Metab. 3 (10), 1290–1301. 10.1038/s42255-021-00483-8 34663974 PMC8889622

[B159] Wlaschek M. Maity P. Makrantonaki E. Scharffetter-Kochanek K. (2021). Connective tissue and fibroblast senescence in skin aging. J. Investigative Dermatology 141 (4), 985–992. 10.1016/j.jid.2020.11.010 33563466

[B160] Woo J. Shin S. Ji H. Ryu D. Cho E. Kim Y. (2022). *Isatis tinctoria* L. Leaf extract inhibits replicative senescence in dermal fibroblasts by regulating mTOR-NF-κB-SASP signaling. Nutrients 14 (9), 1979. 10.3390/nu14091979 35565945 PMC9102489

[B161] Wyles S. P. Carruthers J. D. Dashti P. Yu G. Yap J. Q. Gingery A. (2024). Cellular senescence in human skin aging: leveraging senotherapeutics. Gerontology 70 (1), 7–14. 10.1159/000534756 37879300 PMC10873061

[B162] Xie J. Lin X. Deng X. Tang H. Zou Y. Chen W. (2025). Cancer-associated fibroblast-derived extracellular vesicles: regulators and therapeutic targets in the tumor microenvironment. Cancer Drug Resist 8, 2. 10.20517/cdr.2024.152 39935427 PMC11810458

[B163] Xing H. Lee H. Luo L. Kyriakides T. R. (2020). Extracellular matrix-derived biomaterials in engineering cell function. Biotechnol. Adv. 42, 107421. 10.1016/j.biotechadv.2019.107421 31381963 PMC6995418

[B164] Xu Y. Wei J. Wang W. Mao Z. Wang D. Zhang T. (2025). Oleanolic acid slows down aging through IGF-1 affecting the PI3K/AKT/mTOR signaling pathway. Molecules 30 (3), 740. 10.3390/molecules30030740 39942843 PMC11820160

[B165] Yeerong K. Chantawannakul P. Anuchapreeda S. Rades T. Müllertz A. Chaiyana W. (2024). *Acheta domesticus*: a natural source of anti-skin-aging ingredients for cosmetic applications. Pharm. (Basel) 17 (3), 346. 10.3390/ph17030346 PMC10974149 38543133

[B166] Yokose U. Hachiya A. Sriwiriyanont P. Fujimura T. Visscher M. O. Kitzmiller W. J. (2012). The endogenous protease inhibitor TIMP-1 mediates protection and recovery from cutaneous photodamage. J. Invest Dermatol 132 (12), 2800–2809. 10.1038/jid.2012.204 22718114

[B167] Young A. R. J. Narita M. (2010). Connecting autophagy to senescence in pathophysiology. Curr. Opin. Cell Biol. 22 (2), 234–240. 10.1016/j.ceb.2009.12.005 20045302

[B168] Yu Z. Smith M. J. Siow R. C. M. Liu K. K. (2021). Ageing modulates human dermal fibroblast contractility: quantification using nano-biomechanical testing. Biochim. Biophys. Acta Mol. Cell Res. 1868 (5), 118972. 10.1016/j.bbamcr.2021.118972 33515646

[B169] Zhang H. Xiao X. Wang L. Shi X. Fu N. Wang S. (2024). Human adipose and umbilical cord mesenchymal stem cell-derived extracellular vesicles mitigate photoaging via TIMP1/Notch1. Signal Transduct. Target Ther. 9 (1), 294. 10.1038/s41392-024-01993-z 39472581 PMC11522688

[B170] Zhang J. J. Yu H. Man M. Q. Hu L. (2024). Aging in the dermis: fibroblast senescence and its significance. Aging Cell 23 (2), e14054. 10.1111/acel.14054 38040661 PMC10861215

[B171] Zhang M. Lin Y. Han Z. Huang X. Zhou S. Wang S. (2024). Exploring mechanisms of skin aging: insights for clinical treatment. Front. Immunol. 15, 1421858. 10.3389/fimmu.2024.1421858 39582871 PMC11581952

[B172] Zhao X. Psarianos P. Ghoraie L. S. Yip K. Goldstein D. Gilbert R. (2019). Metabolic regulation of dermal fibroblasts contributes to skin extracellular matrix homeostasis and fibrosis. Nat. Metab. 1 (1), 147–157. 10.1038/s42255-018-0008-5 32694814

